# Improving the predictive performance of binding affinities and poses for protein–cyclic peptide complexes through fine-tuned MM/PBSA(GBSA)-based methods

**DOI:** 10.1093/bib/bbaf632

**Published:** 2025-11-30

**Authors:** Huifeng Zhao, Jianxiang Huang, Gaoqi Weng, Dejun Jiang, Renling Hu, Yu Kang, Tingjun Hou

**Affiliations:** College of Pharmaceutical Sciences, Zhejiang University, Yuhangtang Road 866, Hangzhou, Zhejiang 310058, China; Hangzhou Carbonsilicon AI Technology Co., Ltd, Hangzhou, Zhejiang 310018, China; College of Pharmaceutical Sciences, Zhejiang University, Yuhangtang Road 866, Hangzhou, Zhejiang 310058, China; Medicinal Chemistry and Bioinformatics Center, Shanghai Jiao Tong University School of Medicine, Shanghai 200025, China; College of Pharmaceutical Sciences, Zhejiang University, Yuhangtang Road 866, Hangzhou, Zhejiang 310058, China; Xiangya School of Pharmaceutical Sciences, Central South University, Changsha, Hunan 410004, China; College of Pharmaceutical Sciences, Zhejiang University, Yuhangtang Road 866, Hangzhou, Zhejiang 310058, China; College of Pharmaceutical Sciences, Zhejiang University, Yuhangtang Road 866, Hangzhou, Zhejiang 310058, China; Zhejiang Provincial Key Laboratory for Intelligent Drug Discovery and Development, Jinhua, Zhejiang 321016, China; College of Pharmaceutical Sciences, Zhejiang University, Yuhangtang Road 866, Hangzhou, Zhejiang 310058, China; Zhejiang Provincial Key Laboratory for Intelligent Drug Discovery and Development, Jinhua, Zhejiang 321016, China

**Keywords:** protein–cyclic peptide interaction, MM/PBSA(GBSA), molecular docking, scoring function, binding affinity

## Abstract

Cyclic peptides represent a highly promising class of biopharmaceutical scaffolds. The screening of cyclic peptides against protein targets can be greatly facilitated using computational approaches, especially molecular docking. However, it remains a crucial challenge to accurately predict protein–cyclic peptide (P–cp) interactions employing scoring functions of molecular docking. End-point approaches, such as molecular mechanics generalized Born surface area (MM/GBSA) and molecular mechanics Poisson–Boltzmann surface area (MM/PBSA), provide theoretically more robust frameworks than conventional scoring functions, but their reliability in predicting binding affinities and discriminating native-like binding poses for P–cp complexes remains poorly quantified. Herein, we comprehensively assessed the predictive abilities of MM/PBSA(GBSA) in scoring binding affinities of P–cp complexes and re-ranking their binding poses. The binding affinity scoring ability of MM/PBSA(GBSA) was assessed on a carefully curated dataset consisting of 50 complexes involving P–cp binding affinities, and their re-ranking capability was evaluated on another dataset consisting of the decoys of 81 P–cp complexes. Based on these assessments, we proposed a two-step workflow for predicting P–cp binding affinities. First, we employed the assessed optimal re-ranking method to select the top-1 binding pose; second, we estimated the binding affinity based on the selected top-1 pose using the assessed optimal scoring method. Our proposed workflow, which requires only 3 s for each prediction, achieves binding affinity predictions with a *R_p_* of −0.732 when compared to experimental values, which is twice as high as that of AutoDock CrankPep (*R_p_ =* −0.316). This study emphasizes the necessity of using fine-tuned MM/PBSA(GBSA) methods for predicting P–cp interactions.

## Graphical Abstract

**Graphical Abstract:**
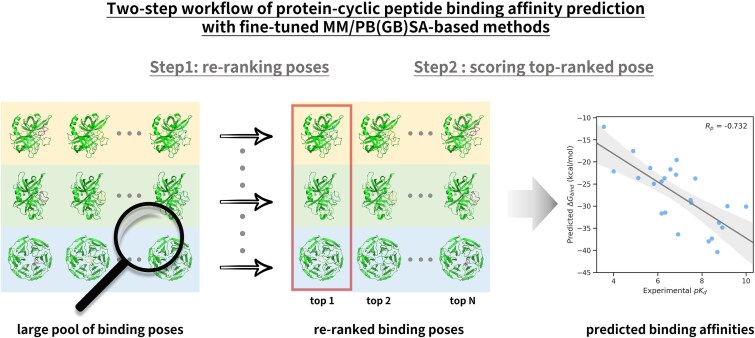

## Introduction

Cyclic peptides, featuring the cyclization of linear peptides, are frequently found in nature and represent a fascinating class of molecules with diverse biological activities, such as anti-infectious agents and hormone signaling for complex biological processes [1]. The biological functions of cyclic peptides inspire their development as scaffolds for biopharmaceutical drugs [2]. To date, over a thousand natural cyclic peptides have been documented, with many of these and their derivatives already successfully developed as pharmaceuticals [3–5]. Cyclic peptides exist in a unique Goldilocks zone between large biologics and small molecules, offering great potential to balance the potency and target specificity of an antibody with the ease-of-administration of a small molecule drug [6–8]. Moreover, cyclic peptides possess favorable pharmacological features when compared with their linear counterparts, including high specificity, high binding affinity, metabolic resistance, and oral bioavailability [7]. Notably, in 2023, three out of the five peptide drugs approved by the FDA were cyclic peptides due to their favorable drug properties: Zilucoplan (Zilbrysq™), Motixafortide (Aphexda™), and Rezzayo (Rezafungin™) [9, 10].

A number of library-based screening methods are available for identifying potent and selective cyclic peptide binders against protein targets, including phage display [11, 12], mRNA display [13] and synthetic combinatorial libraries [14, 15]. Although these methods have successfully discovered cyclic peptide binders, the library size remains significantly limited compared to the vast peptide sequence space, even when considering only the 20 canonical amino acids. Moreover, experimental screening methods often lack detailed insights into the interactions between proteins and cyclic peptides, making them largely empirical endeavors. In contrast, computational design methods for cyclic peptides, such as physics-based methods, heuristic optimization, deep learning, or other computational techniques for searching and ranking solutions, are promising to fully exploit the potential of cyclic peptides due to their high efficiency [16–18]. However, the computational design of strong and specific cyclic peptide inhibitors heavily relies on the reliability of scoring functions, which are crucial for binding affinity prediction and native-like binding pose discrimination of protein–cyclic peptide (P–cp) complexes.

Recently, in 2024, we have systematically compared the performances of 10 commonly used docking programs [19] on the tasks of P–cp interaction prediction, including AutoDock CrankPep (ADCP) [20], Rosetta [16] and eight small molecule–protein docking programs (i.e. AutoDock [21], AutoDock Vina [22], Glide [23], GOLD [24], LeDock [25], rDock [26], MOE [27], and Surflex [28]). Our findings indicate that all 16 scoring functions tested within these docking programs are not satisfactory in terms of estimating binding affinity for P-cp complexes, with poor correlations to the corresponding experimental values, where the best-performing scoring function (MOE@Affinity dG) has a Pearson correlation coefficient (*R_p_*) of only 0.378. These results suggest the limitations of current docking-based scoring functions in P–cp interaction prediction.

End-point approaches, particularly molecular mechanics generalized Born surface area (MM/GBSA) and molecular mechanics Poisson–Boltzmann surface area (MM/PBSA), provide more rigorous theoretical frameworks than conventional scoring functions. These methods have been frequently used as powerful tools to predict the binding affinity and discriminate between “good” and “bad” docking poses [29], and have demonstrated strong performance in predicting protein–protein interactions [30–32] and peptide–protein interactions [33]. Nevertheless, the performance of MM/PBSA(GBSA) methods differs across various systems [34, 35], with accuracy affected by approximations in solvation modeling and entropy estimation [36]. This raises concerns about their accuracy in modeling cyclic peptide–protein interactions, which often involve significant conformational flexibility and solvent effects. Despite these uncertainties, MM/PBSA(GBSA) has been frequently employed in recent studies to rank docking poses of cyclic peptides bound to target proteins [37, 38]. To the best of our knowledge, while systematic evaluation of MM/PBSA(GBSA) methods were conducted for protein–protein systems [30–32] and peptide-protein systems [33], a systematic evaluation of MM/PBSA(GBSA) methods for P–cp systems, including optimized parameter settings and comparisons with docking-based scoring functions, remains lacking.

In this study, we aim to fill this gap by conducting a comprehensive assessment of MM/PBSA(GBSA) methods for both the prediction of binding affinities and the re-ranking performance of the binding poses of P–cp complexes. To comprehensively evaluate the MM/PBSA(GBSA) methods and determine the optimal parameter combination, we systematically investigated key parameters, including force fields, internal dielectric constants (ε_in_), solvation models, and complex structures produced by energy minimizations and sampled with molecular dynamics (MDs) simulations. For binding affinity assessment, MM/PBSA with an interior dielectric ε_in_ = 2, performed on energy-optimized structures with the force field of ff03 and in implicit solvent, achieves the greatest Pearson correlation coefficient (*R_p_*) of −0.545. Our analysis of binding pose re-ranking demonstrated that the optimal MM/PBSA(GBSA) strategies depend on whether P–cp interactions exhibit high or low polarity. For the high polarity subset, MM/PBSA (with ε_in_ = 2), applied to energy-optimized structures under explicit TIP3P solvent using the force field of ff14SBonlysc, achieves the best top-1 success rate (36.4%); while for the low polarity subset, MM/GBSA (with GB^HCT^, ε_in_ = 1), applied to energy-optimized structures under implicit solvent using the force field of ff03, achieves the best top-1 success rate (65.4%). Inspired by these findings, we proposed a two-step workflow that combines the most effective strategies for re-ranking binding poses and predicting binding affinities and applied it to the P–cp binding poses generated by ADCP. This workflow takes on average only 3 s to predict the binding affinity of each P–cp pose and produces binding affinity predictions with an *R_p_* of −0.732 (131.6% better than ADCP) when compared to experimental values. Overall, we believe that the proposed workflow and the comprehensive assessment of MM/PBSA(GBSA) are necessary for the better design of cyclic peptides.

## Materials and Methods

### Datasets

In our previous study [19], we curated a P–cp benchmark dataset named CPSet, which consists of 493 P–cp complexes. For the current study, we filtered CPSet to include only cyclic peptides composed of canonical amino acids, reducing the dataset from 493 to 127 structures. Next, the 127 P–cp complexes were further filtered based on two criteria: availability of experimental binding affinity (K_d_) and no missing residue within 4 Å of the target proteins from the cyclic peptides. Finally, a dataset consisting of 50 P–cp complexes was obtained (denoted as Dataset *I*), which was generated to evaluate the accuracy of binding affinity predictions. The detailed information of Dataset *I,* including peptide chain length, experimental binding affinities and polar interface ratio, is provided in Table S1. Binding affinities are represented as pKd (negative logarithm (base 10) of the dissociation constant: −logKd). The polar interface ratio represents the proportion of the polar interface area within the total binding interface area of P–cp complexes, calculated using the *COCOMAPS* service [39].

To evaluate the pose re-ranking capabilities of MM/PBSA(GBSA), we constructed a re-ranking dataset (Dataset *II*) from the 127 P–cp complexes filtered from CPSet. For each complex, we generated 100 docking poses using ADCP, employing 80 replicas with a partition coefficient of 0.5 to run 10 simulations starting from the helical state and 10 from coil states. Each replica included five million Monte Carlo steps per residue in the cyclic peptide. The “cys” or “cyc” argument was selected to enable the potential for disulfide bonds or backbone amide bonds, respectively, according to the cyclized bonds of the cyclic peptides. The docking poses were then clustered using a native contacts cutoff of 0.8. To assess pose quality, we adopted two metrics: F_nat_, which represents the ratio of native residue–residue contacts within the P–cp interface, and iRMS [40], which measures the root mean square deviation (RMSD) of the interface residue backbones after aligning the receptors of the native and non-native structures. In this study, poses with iRMS ≤2.5 Å and F_nat_ ≥ 0.5 were defined as near-native. For each complex, it was necessary to ensure that at least one of the 100 docking poses qualified as near-native, since re-ranking performance can only be meaningfully evaluated when a correct binding mode is present among the candidates. A total of 81 P–cp complexes satisfied this requirement, forming Dataset *II*.

The same criteria (iRMS ≤2.5 Å and F_nat_ ≥ 0.5) were applied for calculating the success rate. Success rate is the proportion of cases where one or more near-native poses are present among the top-N poses. For example, when MM/GBSA produces hits within the top-3 predictions for 5 out of 100 complexes, the top-3 success rate is 5% (as calculated by 5/100 × 100%).

### Energy minimization and molecular dynamics simulations

Protein Preparation Wizard of the Schrödinger 2020 software [41] was adopted for the preparation of complexes in Dataset *I* and Dataset *II* with default parameters, which added hydrogen atoms and missing residues to the complexes. Six force fields, namely ff14SB [42], ff02 [43], ff03 [44], ff14SBonlysc [45], ff99 [46], and rsff2 [47], were individually assigned to the proteins and cyclic peptides. Specifically, the topology files for each protein and peptide in each complex were generated separately and then merged into a single topology for the complex using the tleap module in AmberTools22 [48]. Subsequently, to prepare solvated P–cp systems, each complex was solvated by a TIP3P water box, with a padding distance of 8 Å along the three dimensions. The simulated systems were further neutralized by adding sodium or chloride ions.

A three-stage energy minimization protocol was performed with Amber22 to relax the systems. Firstly, the protein structure was subjected to 3000 steps of conjugate gradient (CG) after 2000 steps of steepest descent (SD) minimization with a constraint force constant of 50 kcal·mol^−1^·Å^−2^. For the stage 2 minimization, the constraint force constant was decreased to 10 kcal·mol^−1^·Å^−2^ while keeping the other settings identical to the first stage of minimization. For the last stage of minimization, constraints were fully removed for the simulated systems with other computational parameters unchanged from the first two minimizations. In addition, to evaluate the effects of solvent models on the MM/GBSA predictions [49], all complexes underwent energy minimizations (with 2000 steps of SD and 3000 steps of CG) using three different implicit solvent models (GB^HCT^ [50], GB^OBC1^ [5, 1], and GB^OBC2[^^51^^]^). Final structures after all these energy minimizations were adopted for MM/PBSA(GBSA) evaluations.

MD simulations for P-cp complexes were performed with Amber 22, which incorporated the PME algorithm [52] for long-range electrostatic interactions beyond 12 Å. A cutoff distance of 12 Å was also adopted for van der Waals interactions. Bonds involving hydrogen atoms were constrained using the SHAKE algorithm [53], which enabled a 2 fs time step. All MD simulations were started from energy-minimized structures. Each system temperature was increased from 0 to 300 K for 500 ps under canonical ensemble, with the Langevin thermostat [54]. Another equilibration simulation was performed for each system for 1 ns in the NPT ensemble with the Langevin thermostat and the Berendsen barostat to keep the temperature at 300 K and pressure of 1 atm. Finally, each system underwent a production simulation for 6 ns, with the Langevin thermostat (T = 300 K) and the Monte Carlo barostat (P = 1 atm). A total of 250 snapshots were obtained from the last 5 ns production simulations of each system, which were adopted for MM/PBSA(GBSA) evaluations.

### M‌M/PBSA and MM/GBSA calculations

Three GB models were employed for MM/GBSA calculations, including the GB^HCT^, GB^OBC1^, and GB^OBC2^. Considering that there are significant influences of the internal dielectric constant for the solute on the accuracy of MM/PBSA(GBSA) [55], a total of five different internal dielectric constants (i.e. ε_in_ = 1, 2, 4, 6, 8) were adopted for the MM/PBSA(GBSA) calculations. The dielectric constant of the solvent phase was maintained at 80 while the non-polar solvation free energy contributions were calculated via the LCPO algorithm [56] (with *g* of 0.0072 kcal·mol^−1^·Å^−2^ and *b* of 0). Entropy contributions were not included in the MM/PBSA(GBSA) calculations [35, 55, 57]. The predictive accuracy of binding affinities was evaluated using the Pearson correlation coefficient (*R_p_*) to quantify the correlation between computational predictions with experimental measurements.

### Rescoring of decoy binding poses using Rosetta

The “score_jd2” executable within the Rosetta suite was used to independently score the protein, cyclic peptide, and complex. The docking score was obtained by subtracting the sum of the scores for the cyclic peptide and protein from the score of the complex.

## Results

### P-cp binding affinity performance of MM/PBSA(GBSA) on energy-optimized structures under implicit solvent

Energy minimizations were first performed on all 50 P–cp complexes in Dataset *I* (see Materials and Methods) with implicit solvent and six force fields (ff14SB, ff02, ff03, ff14SBonlysc, ff99, and rsff2). Subsequently, binding affinities of P-cp complexes were calculated using MM/PBSA(GBSA) employing three GB models (GB^HCT^, GB^OBC1^, and GB^OBC2^). As demonstrated in Table 1, we observe the best prediction (*R_p_* = −0.545) by MM/PBSA with the ff03 and ε_in_ = 2 (denoted as the optimal scoring method). Additionally, MM/PBSA with ff03 and ε_in_ = 4 also presents a good correlation, which is consistent with our prior study for protein–peptide systems [33], where we also find that MM/PBSA with ε_in_ = 2 yields the best prediction for dataset with relatively short peptide length (5–12 residues). Meanwhile, we observe better performances of MM/PBSA than MM/GBSA, regardless of the adopted interior dielectric constants and force fields. The best prediction of MM/GBSA (*R_p_* = −0.374) is achieved using the GB^OBC1^ model and ε_in_ = 2 with the ff14SB, ff14SBonlysc and ff99. Moreover, the binding affinities results of MM/GBSA employing three distinct GB models show very close prediction performances, with the results with GB^OBC1^ and GB^OBC2^ slightly better than those with the GB^HCT^ model (Table 1).

**Table 1:** Pearson correlation coefficients (*R_p_*) between the experimentally determined pK_d_ and the binding affinities calculated by MM/PBSA or MM/GBSA based on the energy-minimized structures for the entire dataset.

ε^in^	Force field	Implicit solvent*^a^*				Explicit solvent*^b^*			
		GB^HCT^	GB^OBC1^	GB^OBC2^	MM/PBSA	GB^HCT^	GB^OBC1^	GB^OBC2^	MM/PBSA
1	ff14SB	−0.343	−0.364	−0.345	−0.406	−0.263	−0.268	−0.233	−0.371
2		−0.357	−0.374	−0.373	−0.489	−0.346	−0.361	−0.352	−0.470
4		−0.349	−0.356	−0.357	−0.516	−0.375	−0.383	−0.381	−0.506
6		−0.344	−0.349	−0.349	−0.515	−0.382	−0.387	−0.386	**−0.508**
8		−0.342	−0.345	−0.345	−0.487	−0.384	−0.388	−0.388	−0.503
1	ff02	−0.346	−0.349	−0.299	−0.361	−0.338	−0.336	−0.279	−0.444
2		−0.345	−0.352	−0.346	−0.438	−0.373	−0.381	−0.372	−0.489
4		−0.334	−0.337	−0.335	−0.464	−0.382	−0.386	−0.384	−0.492
6		−0.329	−0.331	−0.330	−0.451	−0.383	−0.386	−0.384	−0.486
8		−0.327	−0.328	−0.327	−0.439	−0.383	−0.385	−0.384	−0.480
1	ff03	−0.358	−0.369	−0.340	−0.479	−0.287	−0.285	−0.242	−0.475
2		−0.359	−0.368	−0.364	**−0.545**	−0.346	−0.354	−0.342	−0.484
4		−0.347	−0.351	−0.350	−0.538	−0.362	−0.367	−0.363	−0.472
6		−0.342	−0.344	−0.344	−0.505	−0.366	−0.368	−0.366	−0.455
8		−0.339	−0.341	−0.340	−0.484	−0.367	−0.369	−0.367	−0.445
1	ff14SBonlysc	−0.348	−0.371	−0.354	−0.397	−0.248	−0.255	−0.220	−0.390
2		−0.358	−0.374	−0.375	−0.476	−0.337	−0.354	−0.344	−0.472
4		−0.347	−0.354	−0.356	−0.499	−0.370	−0.379	−0.376	−0.502
6		−0.342	−0.346	−0.347	−0.485	−0.378	−0.383	−0.382	−0.502
8		−0.339	−0.342	−0.343	−0.471	−0.381	−0.385	−0.384	−0.498
1	ff99	−0.342	−0.364	−0.347	−0.384	−0.297	−0.307	−0.273	−0.363
2		−0.357	−0.374	−0.375	−0.475	−0.361	−0.376	−0.367	−0.439
4		−0.349	−0.356	−0.358	−0.505	−0.381	−0.388	−0.386	−0.471
6		−0.344	−0.348	−0.349	−0.491	−0.385	−0.389	−0.388	−0.472
8		−0.341	−0.344	−0.345	−0.476	−0.386	−0.389	−0.388	−0.470
1	rsff2^*^	−0.277	−0.290	−0.265	−0.327	−0.247	−0.250	−0.217	−0.275
2		−0.336	−0.355	−0.354	−0.420	−0.344	−0.360	−0.351	−0.414
4		−0.349	−0.358	−0.360	−0.456	−0.380	−0.389	−0.386	−0.487
6		−0.349	−0.355	−0.356	−0.455	−0.389	−0.394	−0.393	−0.500
8		−0.349	−0.353	−0.354	−0.451	−0.392	−0.396	−0.396	−0.501

*^a^* Using an implicit solvent model. *^b^*Using an explicit solvent model. ^*^rsff2 for peptides in conjunction with ff99SB for proteins.

### P-cp binding affinity performance of MM/PBSA(GBSA) on energy-optimized structures in explicit TIP3P solvent

To examine the effects of solvent models on binding affinity prediction of P–cp complex, P–cp complexes were also subjected to energy minimization in an explicit TIP3P water environment. As demonstrated in Table 1, we observe the best prediction (*R_p_* = −0.508) by MM/PBSA with ε_in_ = 6 and the ff14SB, which is slightly worse than the above best prediction result in implicit solvent (*R_p_* = −0.545). Additionally, we observe obviously better accuracy of MM/PBSA than MM/GBSA when energy minimization is performed with explicit TIP3P solvent, irrespective of the adopted interior dielectric constant and force field, which is consistent with the results obtained from the energy optimized structures under implicit solvent. Furthermore, consistent with previous results in implicit solvent, the binding affinities estimated by MM/GBSA using three different GB models show comparable prediction performance. Moreover, the best predictions of MM/GBSA (*R_p_* = −0.396) are obtained with the rsff2, ε_in_ = 8, and GB^OBC1^ and GB^OBC2^ models, which perform better than the best predictions of MM/GBSA (*R_p_* = −0.374) on the energy-optimized structures under implicit solvent. Overall, the binding affinities estimated by MM/PBSA on the energy-optimized structures under explicit TIP3P solvent are less accurate than those on the energy-optimized structures under implicit solvent for P–cp complexes in Dataset *I*. We speculate that the choice of solvent (i.e. explicit or implicit) has influence on the subsequent polar contribution term calculated by GB or PB methods. Therefore, a systematic comparison is suggested to select the optimal combinations. To sum up, in this specific task, MM/PBSA is much more recommended than MM/GBSA, and the ff03 together with the implicit solvent model, is more recommended in energy minimization for subsequent MM/PBSA calculation of P–cp binding affinities.

### Impact of short MD simulations on the performance of MM/PBSA(GBSA) prediction for P–cp binding affinity

In our prior study, we noticed that short MD simulations did not improve the binding affinity prediction of MM/PBSA(GBSA) for protein–short linear peptide complexes [33] when compared with energy-optimized structures. This raises the question of whether a similar trend holds for cyclic peptides. To address this, we investigate the effects of MD simulations on the binding affinity calculations using MM/PBSA(GBSA) by conducting 5 ns MD simulations (with implicit solvent and explicit TIP3P solvent) on all P–cp complexes in Dataset *I*. The binding affinities were computed utilizing the 5 ns MD trajectories. As demonstrated in Table 2, among the MM/PBSA(GBSA) calculations from the implicit solvent MD simulations, MM/PBSA generally demonstrates superior performance compared to MM/GBSA with three adopted GB models, which is consistent with the results obtained for the energy-optimized structures under implicit solvent (Table 1). Furthermore, the MM/PBSA prediction associated with the ff14SBonlysc and ε_in_ = 8 exhibits the highest correlation (*R_p_* = −0.546), which is very close to the best correlation of *R_p_* = −0.545 with the energy-optimized structures (Table 1). However, among the results obtained with implicit MD simulations, MM/PBSA with ff03 and ff14SBonlysc (with most *R_p_* < −0.45) show better performances compared with other force fields (with *R_p_* > −0.45). In contrast, in results obtained with the structure after energy minimization in implicit solvent, MM/PBSA with all six force fields consistently show good performance, with most *R_p_* < −0.45 (Table 1). These comparisons suggest that, for P–cp complexes, the use of short MD simulations does not necessarily improve binding affinity predictions under the implicit solvent model.

**Table 2:** Pearson correlation coefficients (*R_p_*) between the experimentally determined pK_d_ and the binding affinities calculated by MM/PBSA or MM/GBSA using the structures derived from the 5 ns MD simulations for the entire dataset.

ε_in_	Force field	Implicit solvent^a^				Explicit solvent^b^			
		GB^HCT^	GB^OBC1^	GB^OBC2^	MM/PBSA	GB^HCT^	GB^OBC1^	GB^OBC2^	MM/PBSA
1	ff14SB	−0.247	−0.255	−0.230	−0.307	−0.349	−0.378	−0.358	−0.304
2		−0.270	−0.278	−0.269	−0.393	−0.356	−0.374	−0.374	−0.419
4		−0.274	−0.278	−0.275	−0.435	−0.346	−0.353	−0.354	−0.459
6		−0.275	−0.277	−0.275	−0.435	−0.341	−0.345	−0.346	−0.458
8		−0.275	−0.276	−0.275	−0.431	−0.338	−0.341	−0.342	−0.454
1	ff02	−0.304	−0.303	−0.269	−0.126	−0.357	−0.371	−0.328	−0.443
2		−0.305	−0.306	−0.296	−0.273	−0.321	−0.326	−0.324	−0.468
4		−0.300	−0.300	−0.296	−0.345	−0.297	−0.297	−0.297	−0.444
6		−0.297	−0.297	−0.295	−0.356	−0.289	−0.288	−0.288	−0.421
8		−0.296	−0.296	−0.294	−0.358	−0.284	−0.284	−0.283	−0.406
1	ff03	−0.217	−0.228	−0.200	−0.317	−0.282	−0.284	−0.243	−0.287
2		−0.263	−0.274	−0.264	−0.489	−0.328	−0.338	−0.329	−0.432
4		−0.281	−0.287	−0.283	−0.539	−0.340	−0.345	−0.343	−0.481
6		−0.286	−0.290	−0.287	−0.528	−0.343	−0.346	−0.345	**−0.484**
8		−0.288	−0.291	−0.289	−0.517	−0.344	−0.346	−0.345	−0.483
1	ff14SBonlysc	−0.302	−0.312	−0.289	−0.349	−0.280	−0.300	−0.278	−0.288
2		−0.341	−0.353	−0.345	−0.459	−0.288	−0.304	−0.303	−0.341
4		−0.352	−0.358	−0.355	−0.528	−0.280	−0.287	−0.287	−0.361
6		−0.354	−0.357	−0.356	−0.543	−0.276	−0.280	−0.280	−0.357
8		−0.354	−0.357	−0.356	**−0.546**	−0.273	−0.276	−0.276	−0.352
1	ff99	−0.240	−0.239	−0.212	−0.132	−0.306	−0.320	−0.304	−0.207
2		−0.306	−0.315	−0.304	−0.287	−0.325	−0.337	−0.338	−0.340
4		−0.335	−0.341	−0.337	−0.397	−0.323	−0.328	−0.329	−0.407
6		−0.344	−0.347	−0.345	−0.430	−0.321	−0.324	−0.325	−0.418
8		−0.348	−0.350	−0.349	−0.444	−0.320	−0.322	−0.322	−0.420
1	rsff2^*^	−0.099	−0.103	−0.101	−0.180	−0.223	−0.210	−0.168	−0.157
2		−0.133	−0.142	−0.146	−0.201	−0.262	−0.266	−0.255	−0.257
4		−0.150	−0.155	−0.158	−0.211	−0.271	−0.273	−0.270	−0.309
6		−0.154	−0.158	−0.160	−0.213	−0.272	−0.273	−0.271	−0.315
8		−0.156	−0.159	−0.161	−0.213	−0.272	−0.273	−0.272	−0.314

*^a^* Using an implicit solvent model. *^b^*Using an explicit solvent model. ^*^rsff2 for peptides in conjunction with ff99SB for proteins.

Next, among the MM/PBSA(GBSA) calculations applied to 5 ns explicit TIP3P solvent MD simulations, MM/PBSA generally demonstrates superior performance compared to MM/GBSA employing three GB models, resembling the results obtained with the energy-optimized structures under explicit TIP3P solvent (Table 1). The best prediction based on the explicit-solvent MD simulations is obtained (*R_p_* = −0.484) with ff03 and ε_in_ = 6, which is lower than the best prediction (*R_p_* = −0.508) achieved by MM/PBSA applied to the energy-optimized structures under explicit TIP3P solvent (Table 1). These observations are consistent with previous findings in protein–peptide systems that the MM/PBSA(GBSA) predictions applied to the energy-optimized structures can outperform those from MD simulations [33]. Moreover, MM/PBSA with the explicit TIP3P solvent shows generally worse performances (*R_p_* > −0.45) than MM/PBSA with the implicit solvent model (with most *R_p_* < −0.45).

Taken together, our observations suggest that MM/PBSA(GBSA) results applied to 5 ns implicit solvent and explicit TIP3P solvent MD simulations show drops in prediction capabilities than those performed on the energy-optimized structures. We speculate that the generally worse performance of MM/PBSA(GBSA) results on short MD simulations may stem from force field limitations, especially in representing cyclic peptide flexibility and interactions.

### Binding affinity predicted by MM/PBSA(GBSA) on subsets with high and low polarity

In our previous study, we found that the polarity of protein–peptide binding interface can have a great impact on the predictions of MM/PBSA(GBSA) [30]. To explore the potential optimal parameters for subsets with different polarity features, we classified Dataset *I* into high and low polarity datasets based on the polarity of the complex interface (Table S1). Here, we adopted the polarity definition as the proportion of the polar interface area among the entire interface area. The properties of the P–cp binding interfaces were analyzed by the *COCOMAPS* service [39]. Consistent with our previous study [30], a polarity cutoff of 0.6 was adopted for the classifications of interfaces of high polarity (>0.6) and of low polarity (≤0.6) (Table S1).

**Figure 1:**
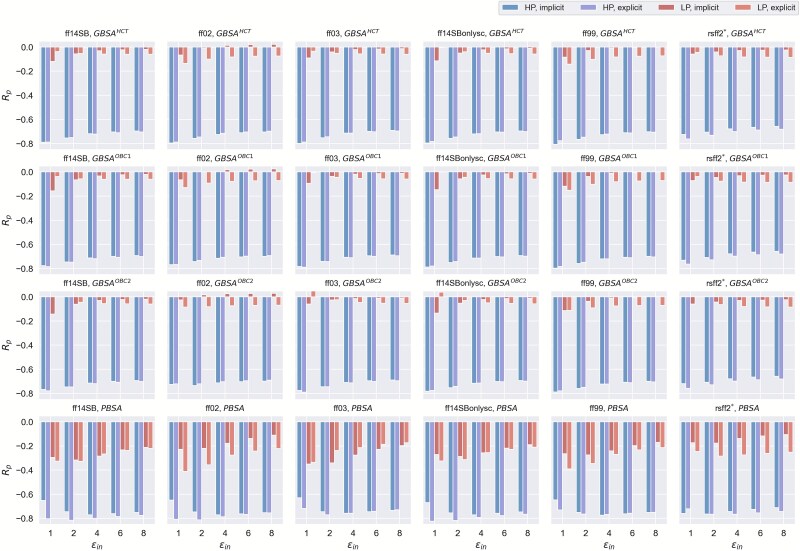
Pearson correlation coefficients (*R_p_*) between the experimentally determined pK_d_ and the predicted binding affinities calculated by MM/PBSA or MM/GBSA using the energy-optimized structures in implicit and explicit solvents for the high polarity (HP) and low polarity (LP) subsets.

Similarly, the performance evaluations of the P–cp pairs in the high and low polarity subsets were performed with MM/PBSA(GBSA) applied to the energy-optimized structures under implicit solvent. Both the high and low polarity subsets contain 25 P–cp complexes each. Interestingly, we observe a quite sound prediction performance on the high polarity dataset with most correlation coefficients ranging from 0.7 to 0.8, while the low polarity dataset exhibits relatively poor correlations (Fig. 1). The best prediction for the high polarity subset is obtained by MM/GBSA with GB^HCT^, the ff99 and ε_in_ = 1 (*R_p_* = 0.807) (Fig. 1 and Table S2), while for the low polarity subset, the best prediction only achieves an *R_p_* of 0.350, obtained from MM/PBSA with the ff03 and ε_in_ = 1 (*R_p_* = 0.350) (Fig. 1 and Table S3).

Next, we explored the influences of solvent models on the prediction capabilities of MM/PBSA(GBSA) on the high and low polarity subsets. Interestingly, in contrast to the entire dataset *I* results in Table 1 where implicit solvents showed more accurate predictions (Table 1), we observe an improvement in prediction accuracy for both subsets when using energy-optimized structures under explicit TIP3P solvents compared to those in implicit solvents (Fig.1, Tables S2 and S3). As illustrated in Fig. S1, while the implicit solvent performs slightly better on the entire dataset *I*, explicit solvent models produce stronger correlations for both high and low polarity subsets. This observation may be related to the fact that explicit TIP3P solvents can better capture electrostatic and hydrogen-bonding interactions at high polarity interfaces, as well as hydrophobic interactions at low polarity interfaces. By contrast, for the entire Dataset *I*, the implicit solvents’ generalized treatment of solvation effects appears to provide more consistent performance. Furthermore, we investigated the effects of MD simulations on the binding affinity predictions of MM/PBSA(GBSA) for the high and low polarity subsets. Consistent with previous observations that MD simulations do not contribute to the improved predictions of MM/PBSA(GBSA) (Tables 1 and 2), the same conclusion can be made for both the high and low polarity subsets (Tables S4, S5 and Fig.  S3).

### Binding affinity predicted by MM/PBSA(GBSA) on subsets with short and long peptide length

**Figure 2:**
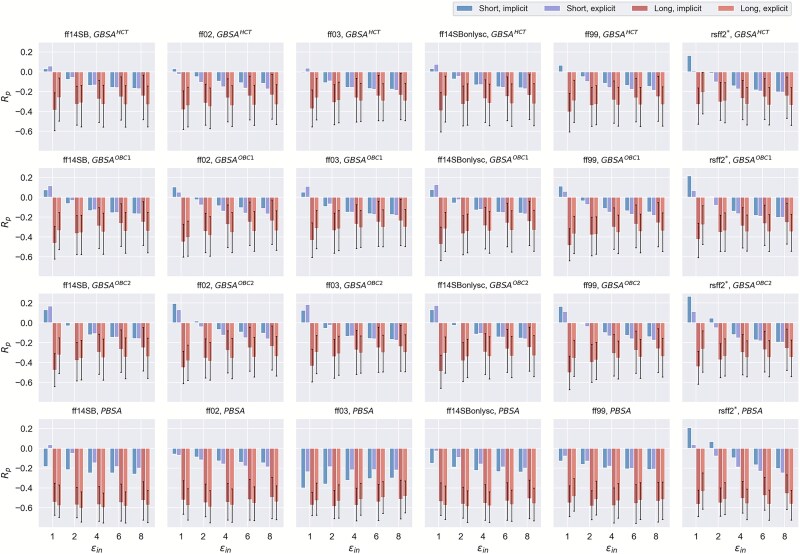
Pearson correlation coefficients (*R_p_*) between the experimentally determined pK_d_ and the predicted binding affinities calculated by MM/PBSA or MM/GBSA using the energy-optimized structures in implicit and explicit solvents for the “short” and “long” subsets. Error bars for the “long” subset represent 90% confidence intervals obtained from 100 bootstrapped resampling (N = 18).

Besides the polarity feature, peptide chain length is another feature we would like to explore to divide Dataset *I* into subsets with different peptide chain lengths and further evaluate the performance of MM/PBSA(GBSA) on these subsets (Table S1 and Fig.  S4). We divided the dataset into a “short” subset (cyclic peptide length ≤ 10 aa) and a “long” subset (cyclic peptide length > 10 aa), resulting 18 and 32 P–cp complexes in the “short” and “long” subsets, respectively. To account for the unequal sample sizes between the two subsets, we performed a bootstrap resampling analysis by repeatedly sampling 18 complexes from the long subset 100 times to match the size of the short subset. Then, *R_p_* were calculated for each resampling to ensure that the comparison between subsets was statistically balanced. The performance evaluations of the P–cp pairs in the short and long subsets were first conducted with the energy-optimized structures under the implicit solvent model. Interestingly, significantly better performances are observed on the long subset than on the short subset (Fig. 2). Meanwhile, MM/PBSA produces better predictions than MM/GBSA on both the long and short subsets with three different GB models. The best prediction for the short subset is achieved by MM/PBSA with ε_in_ = 1 and the ff03 (*R_p_* = −0.400) (Fig. 2 and Table S6) while the best prediction for the long subset is obtained using MM/PBSA with ε_in_ = 2 and the ff03 (*R_p_* = −0.587 ± 0.085) (Fig. 2 and Table S7).

Next, we studied the influences of solvent models on the prediction capabilities of MM/PBSA(GBSA) for the short and long subsets. Interestingly, while the prediction performances on the energy-optimized structures for the short subset under explicit TIP3P solvents are decreased compared with those under the implicit solvent (Table S6), the prediction performances of the energy-optimized structures for the long subset under explicit TIP3P solvents are improved in comparison with those under the implicit solvent (Table S7). Again, 5 ns MD simulations do not improve the prediction capabilities of MM/PBSA(GBSA) on both the short and long subsets (Tables S8 and S9).

### Performance of MM/PBSA(GBSA) in re-ranking binding poses

We set out to assess the performance of MM/PBSA(GBSA) in re-ranking binding poses. Specifically, we assessed the re-ranking capabilities of MM/PBSA(GBSA) for docking on Dataset *II*, consisting of the docking poses generated for the 81 P–cp complexes by ADCP (see Materials and Methods). For comparison, we also employed the scoring functions in ADCP and “ref2015” of the Rosetta packages. According to our previous study [19], “ref2015” in Rosetta shows the best performance in selecting optimal conformations. We applied the MM/PBSA(GBSA) calculations to the energy-optimized structures with two best-performing force fields (ff03 and ff14SBonlysc) under both implicit and explicit TIP3P solvent models. The re-ranking results for all of the 81 P–cp complexes are summarized in Tables S10–S13.

**Figure 3:**
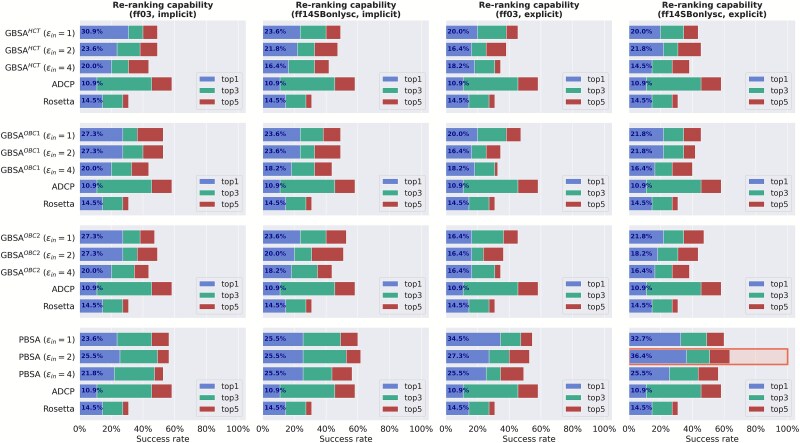
Success rates of ADCP, Rosetta and different MM/PBSA and MM/GBSA protocols for the high polarity subset. The highest top-1 success rate is highlighted by a rectangle.

**Figure 4:**
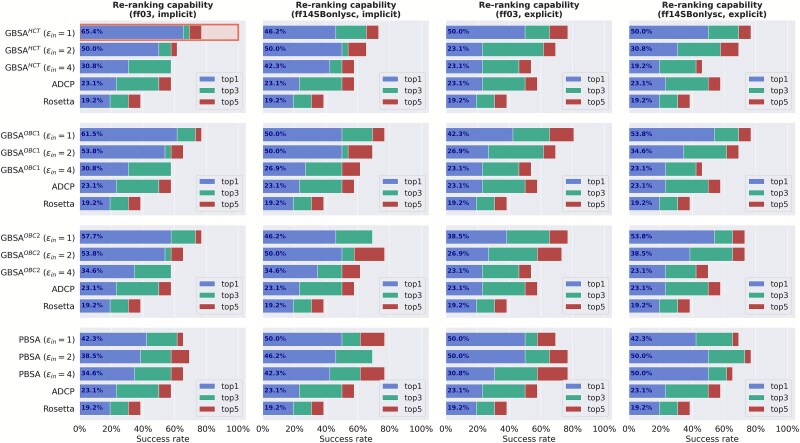
Success rates of ADCP, Rosetta and different MM/PBSA and MM/GBSA protocols for the low polarity subset. The highest top-1 success rate is highlighted by a rectangle.

To further find the optimal parameters of MM/PBSA(GBSA) on different polarity subsets, we calculated the success rates of top-N (N = 1, 3, and 5) using ADCP, Rosetta, MM/PBSA(GBSA) on high and low polarity subsets (derived from Dataset *II*). MM/PBSA with the ff14SBonlysc and ε_in_ = 2 on the energy-optimized structures under explicit TIP3P solvent shows the best top-1 success rate (36.4%) for the high polarity subset (Fig. 3). For the low polarity subset, the best re-ranking performance is achieved by MM/GBSA (GB^HCT^) with the ff03 and ε_in_ = 1 on the energy-optimized structures under implicit solvent, with a top-1 success rate of 65.4% (Fig. 4). For clarity, these two strategies are denoted as the optimal re-ranking methods. Interestingly, MM/PBSA performs better for high-polarity interfaces, while MM/GBSA performs better for low-polarity interfaces. This may be primarily due to the more accurate treatment of long-range electrostatics by the PB method compared to the GB method used in MM/GBSA. As a result, MM/PBSA is theoretically more sensitive to variations in charged and highly polar residue interactions, which are often dominant in high-polarity interfaces. Meanwhile, MM/PBSA may be better at handling solvent polarization and dielectric discontinuities, allowing it to accurately model electrostatic desolvation penalties at polar interfaces. On both subsets, the re-ranking capacities of these specific MM/PBSA(GBSA)-based methods outperform ADCP and Rosetta (Fig. 3 and Fig. 4).

We also analyzed the top-*N* success rates on the short (Fig. S5) and long subsets (Fig. S6). As displayed in Fig. S5 and S6, the top-1 success rates are 51.2% (MM/GB^HCT^SA, ε_in_ = 1, with the energy-optimized structures using the ff03 and implicit solvent) on the short subset and 47.4% (MM/PBSA, ε_in_ = 2, with the energy-optimized structures using the ff14SBonlysc and explicit TIP3P solvent) on the long subset. These performances also surpass those of ADCP and Rosetta. The above observations emphasize the necessity of adopting the MM/PBSA(GBSA) methods for cyclic peptide-related tasks and the rationality of adopting different MM/PBSA(GBSA) strategies on different subsets.

### A two-step workflow that improves binding affinity prediction for P–cp complexes

**Figure 5:**
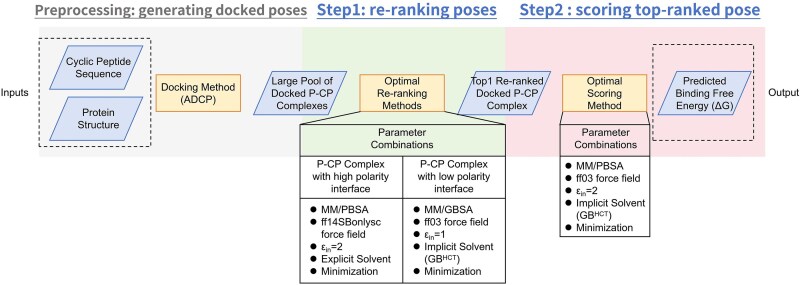
A two-step workflow for improving binding affinity prediction of protein-cyclic peptide (P-cp) complexes. Step 1 involves re-ranking docked P-cp poses using polarity-specific optimal methods: MM/PBSA with the ff14SBonlysc force field and ε_in_ = 2 in explicit TIP3P solvent for high-polarity interfaces, and MM/GBSA with GB^HCT^, the ff03 force field and ε_in_ = 1 in implicit solvent for low-polarity interfaces. Step 2 uses the top-1 re-ranked pose for energy minimization under implicit solvent (ff03, ε_in_ = 2) and calculates the binding affinity with MM/PBSA to obtain the predicted binding free energy.

**Figure 6:**
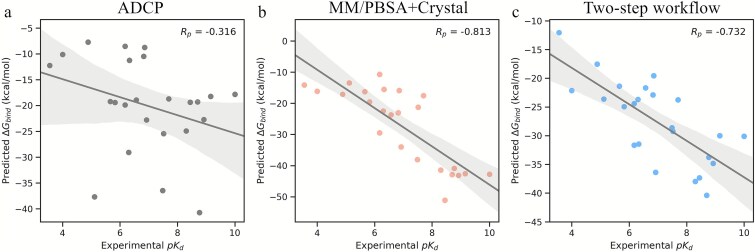
(a) Correlations of the experimental *pK_d_* with predicted binding affinity of the top-1 complex structure evaluated by ADCP. (b) Correlations of the experimental *pK_d_* with predicted binding affinity of the energy-optimized crystal complexes in implicit solvent evaluated by MM/PBSA with ff03 and ε_in_ = 2. (c) Correlations of the experimental *pK_d_* with predicted binding affinity of the energy-optimized re-ranked (using the two-step workflow) top-1 pose in implicit solvent evaluated by MM/PBSA with ff03 and ε_in_ = 2.

Based on the above-mentioned assessments, we designed a two-step workflow for binding poses re-ranking and binding affinity prediction of P–cp complexes (Fig. 5). We screened data from Dataset *II*, consisting of docking poses generated by ADCP, and picked out those with experimental binding affinities. Subsequently, a decoy subset of 24 P–cp complexes was built to evaluate the performance of our two-step workflow (Fig. S7). First, we assessed the binding affinity prediction ability of ADCP, as a baseline, by comparing the predicted binding affinities of the top-ranked poses generated by ADCP with the experimental binding affinities, and an *R_p_* of −0.316 was obtained (Fig. 6a), suggesting a poor predictive accuracy for binding affinity. Second, we applied MM/PBSA (ε_in_ = 2) to the energy-optimized structures under the implicit solvent with the ff03 on 24 P–cp complexes, namely, the optimal scoring method. As expected, a much higher *R_p_* of −0.813 is obtained (Fig. 6b), which is supposed to serve as an upper limit for our carefully assessed MM/PBSA(GBSA)-based method for P–cp binding affinity prediction.

Given that different strategies work best for different subsets (see Fig. 3 and Fig. 4), we decided to use a divide-and-conquer two-step workflow for the re-ranking of the 24 P–cp decoys (Fig. 5), thus allowing us to assign specific re-ranking methods tailored to the characteristics of different P–cp pairs. Here, during the first step, we re-ranked the decoy poses of the 24 complexes in the decoy subset with high and low interface polarity. Specifically, MM/PBSA with the ff14SBonlysc and ε_in_ = 2 in explicit TIP3P solvent were adopted for the complexes with high polarity interface and MM/GBSA with GB^HCT^, the ff03 and ε_in_ = 1 in implicit solvent for the complexes with low polarity interface (i.e. the optimal re-ranking methods). During the second step, the top-1 poses of the re-ranked decoys were used for energy minimization in the implicit solvent with ff03 and ε_in_ = 2, and the binding affinity calculations were performed for the energy-optimized top-1 poses using MM/PBSA (i.e. the optimal scoring method). Strikingly, the predicted binding affinities show a strong correlation with the experimental values (*R_p_* = −0.732) (Fig. 6c), which is close to the upper limit *R_p_* = −0.813, and is 131.6% higher than that of the state-of-the-art ADCP (*R_p_* of −0.316). Moreover, this two-step workflow requires on average 3 s to predict the binding affinity for each P–cp pose (tested on a workstation with an AMD EPYC 7 K62 48-Core Processor using 24 CPU threads and an NVIDIA GeForce RTX 4090 GPU with 24 GB memory), achieving an excellent balance between accuracy and efficiency.

## Conclusion

In this study, we systematically evaluated the performance of MM/PBSA(GBSA) methods for predicting the binding affinities and re-ranking the binding poses of P–cp complexes. Using a curated dataset of 50 P–cp crystal structures (Dataset *I*), we found that MM/PBSA with ε_in_ = 2 applied to the energy-optimized structures under implicit solvent using the ff03 predicted the binding affinities best (*R_p_* = −0.545). Our results further suggest that the contribution from MD simulations sampling is not significant for the MM/PBSA(GBSA) predictions of P–cp systems.

Next, we evaluated the re-ranking capabilities of MM/PBSA(GBSA) on Dataset *II*, which consists of the docking poses generated for the 81 P–cp complexes by ADCP. Dataset *II* was divided into high polarity and low polarity subsets to further explore the optimal parameters of MM/PBSA(GBSA). Detailed analyses reveal that for the high polarity subset, a combination of MM/PBSA with ε_in_ = 2 applied to the energy-optimized structures under explicit TIP3P using the ff14SBonlysc produces the highest top-1 success rate (36.4%); while a combination of MM/GBSA (GB^HCT^) with ε_in_ = 1 applied to the energy-optimized structures under implicit solvent using the ff03 yields the best top-1 success rate (65.4%) for the low polarity subset.

Based on these observations, we proposed a two-step workflow that integrates the optimal re-ranking and re-scoring strategies identified above and applied to a subset of 24 P–cp complexes. This workflow achieved a significantly improved correlation with experimental binding affinities (*R_p_* = −0.732), yielding a 131.6% improvement over the existing state-of-the-art method, ADCP. Notably, this two-step workflow requires on average only 3 s for each P–cp prediction, thus offering an effective balance between accuracy and efficiency.

To sum up, our findings demonstrate that with appropriate parameter tuning, MM/PBSA(GBSA) methods can provide a reliable framework for scoring P–cp complexes. Compared with the existing state-of-the-art scoring functions used in P–cp docking tools (e.g. ADCP and Rosetta), MM/PBSA(GBSA) can achieve better accuracy for both predicting the binding affinities and identifying correct binding poses for P–cp complexes. Our study emphasizes the potential of MM/PBS(GBSA) methods for the development of potent and specific cyclic peptide binders. The two-step workflow proposed in this study is optimized for virtual screening and affinity ranking of cyclic peptides. Finally, the curated benchmark datasets established in this work may serve as valuable resources for the further development and validation of next-generation computational models, such as machine learning approaches tailored to P-cp interactions.

**Key Points**

- Two benchmark datasets of protein–cyclic peptide complexes were established to evaluate the binding affinity prediction capabilities and the re-ranking performance of various MM/PBSA(GBSA) protocols.
- A systematic assessment of multiple MM/PBSA(GBSA) protocols was conducted to identify the most effective approach to predict binding affinities and discriminate native-like binding poses for protein–cyclic peptide systems complexes.
- We further proposed a two-step workflow for predicting protein–cyclic peptide binding affinities within only a few seconds, which achieves an excellent Pearson correlation coefficient of −0.732 with experimental data, significantly higher than the state-of-the-art (i.e. ADCP).

## Supplementary Material

SI_bbaf632

## Data Availability

The data is freely available at https://github.com/huifengzhao/MM_PBGBSA-CP.

## Notes

Basic information of Dataset *I* (PDB IDs included); Pearson’s correlation coefficients of various MM/PBSA(GBSA) methods on four subsets of Dataset *I*; ranks given by ADCP, Rosetta and various MM/PBSA(GBSA) methods for Dataset *II*; comparison of Pearson’s correlation coefficients between energy-optimized and 5 ns MD-simulated structures on high- and low-polarity subsets; scatter plot of interface polarity versus cyclic peptide length for Dataset *I* and Dataset *II*; success rates of ADCP, Rosetta and various MM/PBSA(GBSA) methods on two subsets of Dataset *II*; distributions of cyclic peptide length and interface polarity in Dataset *I* and Dataset *II*.
