# Supplementary material for: Improving the predictive performance of binding affinities and poses for protein–cyclic peptide complexes through fine-tuned MM/PBSA(GBSA)-based methods
Source: Brief Bioinform. 2025 Nov 30;26(6):bbaf632. doi: 10.1093/bib/bbaf632 (PMC12665037; doi:10.1093/bib/bbaf632)
Supplement: SI_bbaf632 [file si_bbaf632.docx]

**Supporting Information**

**Improving the predictive performance of binding affinities and poses for protein-cyclic peptide complexes through fine-tuned MM/PBSA(GBSA)-based methods**

Huifeng Zhao^1,2,#^, Jianxiang Huang^1,3,#^, Gaoqi Weng^1^, Dejun Jiang^4^, Renling Hu^1^, Yu Kang^1,5,^*, Tingjun Hou^1,5,^*

^1^College of Pharmaceutical Sciences, Zhejiang University, Hangzhou 310058, Zhejiang, China

^2^Hangzhou Carbonsilicon AI Technology Co., Ltd, Hangzhou 310018, Zhejiang, China

^3^Medicinal Chemistry and Bioinformatics Center, Shanghai Jiao Tong University School of Medicine, Shanghai, 200025, China

^4^Xiangya School of Pharmaceutical Sciences, Central South University, Changsha 410004, Hunan, China

^5^Zhejiang Provincial Key Laboratory for Intelligent Drug Discovery and Development, Jinhua 321016, Zhejiang, China

#Equivalent authors

***Corresponding authors**

**Tingjun Hou**

E-mail: tingjunhou@zju.edu.cn

**Yu Kang**

E-mail: yukang@zju.edu.cn

**Table S1.** Some basic information and the experimental pKd values for the dataset *I*

| PDB code | Chain id | Peptide length | Polar interface (%) | K_d_ (M) | pK_d_ | Chain type |
| --- | --- | --- | --- | --- | --- | --- |
| 1G9I | E:I*^a^* | 22 | 58.64 | 1.20E-07 | 6.92 | SS*3*^b^* |
| 1SFI | A:I | 14 | 61.97 | 1.00E-10 | 10.00 | BB+SS*^c^* |
| 1SLD | B:P | 6 | 73.42 | 2.70E-07 | 6.57 | SS*1 |
| 1SLE | BD:M | 8 | 77.40 | 6.70E-07 | 6.17 | SS*1 |
| 1SMF | E:I | 9 | 66.97 | 1.20E-07 | 6.92 | SS*1 |
| 1VWB | B:P | 6 | 73.17 | 2.00E-08 | 7.70 | SS*1 |
| 1VWD | B:P | 6 | 71.95 | 2.20E-06 | 5.66 | SS*1 |
| 1VWN | B:P | 6 | 73.35 | 1.50E-06 | 5.82 | SS*1 |
| 1VWO | B:P | 8 | 74.40 | 2.30E-06 | 5.64 | SS*1 |
| 1VWP | B:P | 8 | 74.30 | 2.30E-06 | 5.64 | SS*1 |
| 1YF4 | A:B | 9 | 50.68 | 5.00E-09 | 8.30 | SS*1 |
| 2BR8 | AB:F | 16 | 67.27 | 3.26E-08 | 7.49 | SS*2 |
| 2NWN | A:B | 12 | 51.01 | 5.00E-07 | 6.30 | SS*1 |
| 2UZ6 | AB:K | 16 | 66.76 | 1.70E-09 | 8.77 | SS*2 |
| 3G5Y | AB:E | 16 | 48.02 | 3.50E-08 | 7.46 | SS*1 |
| 3OY5 | U:P | 15 | 51.96 | 5.40E-06 | 5.27 | SS*1 |
| 3P8F | A:I | 14 | 65.48 | 9.20E-10 | 9.04 | BB+SS |
| 3PP4 | HL:P | 24 | 54.65 | 7.00E-06 | 5.15 | SS*1 |
| 4GLY | A:B | 13 | 48.12 | 7.70E-06 | 5.11 | SS*2 |
| 4GW1 | AB:E | 12 | 57.80 | 4.30E-07 | 6.37 | SS*1 |
| 4GW5 | AB:E | 12 | 65.07 | 3.50E-06 | 5.46 | SS*1 |
| 4IB5 | A:D | 13 | 69.26 | 5.60E-07 | 6.25 | SS*1 |
| 4K1E | A:B | 14 | 65.55 | 3.59E-09 | 8.44 | BB+SS |
| 4M1D | HL:P | 14 | 64.09 | 2.30E-08 | 7.64 | SS*1 |
| 4W50 | A:E | 12 | 66.77 | 1.40E-06 | 5.85 | SS*1 |
| 4X1Q | U:P | 10 | 56.36 | 5.04E-06 | 5.30 | SS*1 |
| 4Z0D | A:C | 13 | 62.38 | 5.00E-06 | 5.30 | SS*1 |
| 4ZHL | U:P | 10 | 59.90 | 3.40E-07 | 6.47 | SS*1 |
| 5EOC | HL:P | 13 | 61.52 | 3.03E-08 | 7.52 | SS*1 |
| 5EUK | AB:E | 12 | 56.84 | 5.00E-05 | 4.30 | SS*1 |
| 5F88 | AB:E | 12 | 59.46 | 5.00E-05 | 4.30 | SS*1 |
| 5FF6 | AB:E | 12 | 57.98 | 9.50E-06 | 5.02 | SS*1 |
| 5H5Q | A:B | 13 | 53.79 | 1.80E-06 | 5.74 | SS*1 |
| 5H5R | A:B | 13 | 51.70 | 6.10E-07 | 6.21 | SS*1 |
| 5TH2 | AB:E | 12 | 58.57 | 3.00E-05 | 4.52 | SS*1 |
| 5VB9 | A:C | 15 | 67.69 | 1.30E-06 | 5.89 | SS*1 |
| 5XCO | A:B | 19 | 63.32 | 8.90E-09 | 8.05 | SS*1 |
| 5XN3 | A:B | 8 | 49.33 | 6.71E-07 | 6.17 | BB |
| 6BVH | A:I | 14 | 58.18 | 7.00E-10 | 9.15 | BB+SS |
| 6D3X | AB:C | 14 | 66.75 | 5.10E-11 | 10.29 | BB+SS |
| 6D3Y | A:C | 14 | 58.28 | 2.00E-09 | 8.70 | BB+SS |
| 6D3Z | A:C | 14 | 62.30 | 1.20E-09 | 8.92 | BB+SS |
| 6D40 | A:C | 14 | 64.70 | 1.40E-10 | 9.85 | BB+SS |
| 6O21 | A:B | 11 | 68.48 | 3.48E-09 | 8.46 | SS*1 |
| 7K2E | A:P | 7 | 48.60 | 4.70E-07 | 6.33 | BB |
| 7K2F | A:C | 7 | 48.76 | 1.00E-04 | 4.00 | BB |
| 7K2G | B:P | 7 | 47.51 | 2.80E-04 | 3.55 | BB |
| 7K2H | B:P | 7 | 50.96 | 1.40E-07 | 6.85 | BB |
| 7K2I | B:P | 7 | 52.40 | 1.30E-05 | 4.89 | BB |
| 7K2M | A:P | 7 | 50.72 | 1.50E-07 | 6.82 | BB |

*^a^* The letter(s) preceding ":" denote protein chains, while the letter following ":" indicates the cyclic peptide chain. *^b^* "SS" denotes cyclization via disulfide bonds, with the number after "*" indicating the count of such bonds. *^c^* "BB" signifies cyclization through amide bonds connecting the head and tail of the backbone.

**Table S2.** Pearson correlation coefficients (*R_p_*) between experimentally determined pK_d_ and the binding affinities calculated by MM/PBSA or MM/GBSA using energy minimized structures for the high polarity subset

| εin | Force field | Implicit solvent | | | | Explicit solvent | | | |
| --- | --- | --- | --- | --- | --- | --- | --- | --- | --- |
|  |  | GBHCT | GBOBC1 | GBOBC2 | PBSA | GBHCT | GBOBC1 | GBOBC2 | PBSA |
| 1 | ff14SB | -0.789 | -0.776 | -0.766 | -0.652 | -0.786 | -0.784 | -0.778 | -0.802 |
| 2 |  | -0.753 | -0.746 | -0.747 | -0.745 | -0.750 | -0.747 | -0.746 | -0.815 |
| 4 |  | -0.718 | -0.713 | -0.714 | -0.771 | -0.721 | -0.718 | -0.718 | -0.800 |
| 6 |  | -0.703 | -0.700 | -0.700 | -0.761 | -0.710 | -0.707 | -0.707 | -0.786 |
| 8 |  | -0.696 | -0.693 | -0.693 | -0.751 | -0.703 | -0.702 | -0.702 | -0.775 |
| 1 | ff02 | -0.795 | -0.769 | -0.726 | -0.649 | -0.786 | -0.767 | -0.720 | -0.808 |
| 2 |  | -0.757 | -0.743 | -0.735 | -0.747 | -0.745 | -0.734 | -0.721 | -0.813 |
| 4 |  | -0.724 | -0.715 | -0.712 | -0.771 | -0.715 | -0.708 | -0.702 | -0.787 |
| 6 |  | -0.711 | -0.705 | -0.703 | -0.762 | -0.703 | -0.698 | -0.695 | -0.768 |
| 8 |  | -0.704 | -0.699 | -0.698 | -0.753 | -0.697 | -0.693 | -0.691 | -0.755 |
| 1 | ff03 | -0.798 | -0.782 | -0.776 | -0.630 | -0.786 | -0.789 | -0.790 | -0.718 |
| 2 |  | -0.751 | -0.742 | -0.744 | -0.745 | -0.744 | -0.742 | -0.745 | -0.771 |
| 4 |  | -0.713 | -0.707 | -0.709 | -0.759 | -0.713 | -0.711 | -0.713 | -0.758 |
| 6 |  | -0.699 | -0.694 | -0.695 | -0.744 | -0.702 | -0.700 | -0.701 | -0.741 |
| 8 |  | -0.691 | -0.688 | -0.689 | -0.733 | -0.696 | -0.694 | -0.695 | -0.730 |
| 1 | ff14SBonlysc | -0.795 | -0.788 | -0.781 | -0.670 | -0.782 | -0.779 | -0.775 | -0.823 |
| 2 |  | -0.757 | -0.751 | -0.753 | -0.754 | -0.746 | -0.742 | -0.742 | -0.818 |
| 4 |  | -0.719 | -0.714 | -0.716 | -0.770 | -0.717 | -0.714 | -0.714 | -0.794 |
| 6 |  | -0.704 | -0.700 | -0.701 | -0.758 | -0.706 | -0.703 | -0.703 | -0.777 |
| 8 |  | -0.696 | -0.693 | -0.693 | -0.746 | -0.700 | -0.698 | -0.698 | -0.765 |
| 1 | ff99 | -0.807 | -0.797 | -0.788 | -0.647 | -0.776 | -0.782 | -0.779 | -0.731 |
| 2 |  | -0.765 | -0.758 | -0.759 | -0.751 | -0.748 | -0.749 | -0.749 | -0.765 |
| 4 |  | -0.726 | -0.721 | -0.722 | -0.774 | -0.722 | -0.721 | -0.721 | -0.767 |
| 6 |  | -0.710 | -0.706 | -0.707 | -0.762 | -0.711 | -0.710 | -0.710 | -0.758 |
| 8 |  | -0.702 | -0.699 | -0.700 | -0.751 | -0.705 | -0.704 | -0.705 | -0.750 |
| 1 | rsff2* | -0.725 | -0.732 | -0.718 | -0.759 | -0.761 | -0.763 | -0.757 | -0.720 |
| 2 |  | -0.704 | -0.709 | -0.708 | -0.764 | -0.730 | -0.729 | -0.729 | -0.766 |
| 4 |  | -0.677 | -0.678 | -0.678 | -0.743 | -0.700 | -0.698 | -0.698 | -0.766 |
| 6 |  | -0.665 | -0.664 | -0.665 | -0.725 | -0.688 | -0.686 | -0.686 | -0.754 |
| 8 |  | -0.658 | -0.657 | -0.658 | -0.712 | -0.681 | -0.679 | -0.680 | -0.743 |

**Table S3.** Pearson correlation coefficients (*R_p_*) between experimentally determined pKd and the binding affinities calculated by MM/PBSA or MM/GBSA using energy minimized structures for the low polarity subset

| εin | Force field | Implicit solvent | | | | Explicit solvent | | | |
| --- | --- | --- | --- | --- | --- | --- | --- | --- | --- |
|  |  | GBHCT | GBOBC1 | GBOBC2 | PBSA | GBHCT | GBOBC1 | GBOBC2 | PBSA |
| 1 | ff14SB | -0.120 | -0.156 | -0.143 | -0.295 | -0.037 | -0.038 | 0.000 | -0.326 |
| 2 |  | -0.056 | -0.065 | -0.061 | -0.316 | -0.054 | -0.058 | -0.045 | -0.327 |
| 4 |  | -0.030 | -0.032 | -0.030 | -0.284 | -0.058 | -0.060 | -0.055 | -0.266 |
| 6 |  | -0.023 | -0.024 | -0.022 | -0.232 | -0.059 | -0.060 | -0.057 | -0.237 |
| 8 |  | -0.019 | -0.020 | -0.019 | -0.213 | -0.059 | -0.060 | -0.058 | -0.219 |
| 1 | ff02 | -0.067 | -0.065 | -0.026 | -0.227 | -0.136 | -0.129 | -0.085 | -0.411 |
| 2 |  | -0.009 | 0.000 | 0.017 | -0.220 | -0.099 | -0.092 | -0.081 | -0.356 |
| 4 |  | 0.013 | 0.019 | 0.026 | -0.178 | -0.082 | -0.077 | -0.073 | -0.276 |
| 6 |  | 0.020 | 0.023 | 0.028 | -0.138 | -0.076 | -0.073 | -0.071 | -0.242 |
| 8 |  | 0.023 | 0.025 | 0.029 | -0.111 | -0.074 | -0.071 | -0.069 | -0.222 |
| 1 | ff03 | -0.090 | -0.093 | -0.059 | -0.350 | -0.033 | -0.006 | 0.058 | -0.335 |
| 2 |  | -0.040 | -0.037 | -0.025 | -0.340 | -0.052 | -0.045 | -0.024 | -0.238 |
| 4 |  | -0.021 | -0.019 | -0.014 | -0.277 | -0.057 | -0.055 | -0.047 | -0.213 |
| 6 |  | -0.016 | -0.014 | -0.011 | -0.229 | -0.059 | -0.057 | -0.052 | -0.186 |
| 8 |  | -0.013 | -0.012 | -0.010 | -0.197 | -0.059 | -0.058 | -0.055 | -0.174 |
| 1 | ff14SBonlysc | -0.115 | -0.148 | -0.134 | -0.271 | -0.003 | -0.001 | 0.039 | -0.325 |
| 2 |  | -0.050 | -0.056 | -0.053 | -0.288 | -0.039 | -0.044 | -0.030 | -0.313 |
| 4 |  | -0.023 | -0.024 | -0.022 | -0.257 | -0.052 | -0.055 | -0.050 | -0.256 |
| 6 |  | -0.016 | -0.016 | -0.015 | -0.218 | -0.056 | -0.058 | -0.055 | -0.227 |
| 8 |  | -0.012 | -0.012 | -0.011 | -0.190 | -0.057 | -0.059 | -0.057 | -0.211 |
| 1 | ff99 | -0.084 | -0.119 | -0.114 | -0.265 | -0.141 | -0.152 | -0.113 | -0.390 |
| 2 |  | -0.029 | -0.038 | -0.037 | -0.275 | -0.102 | -0.102 | -0.091 | -0.345 |
| 4 |  | -0.008 | -0.011 | -0.010 | -0.240 | -0.082 | -0.081 | -0.076 | -0.270 |
| 6 |  | -0.002 | -0.004 | -0.003 | -0.198 | -0.076 | -0.075 | -0.072 | -0.233 |
| 8 |  | 0.000 | 0.000 | 0.000 | -0.170 | -0.072 | -0.072 | -0.070 | -0.214 |
| 1 | rsff2* | -0.058 | -0.072 | -0.059 | -0.173 | -0.043 | -0.038 | 0.000 | -0.245 |
| 2 |  | -0.040 | -0.046 | -0.044 | -0.176 | -0.072 | -0.076 | -0.064 | -0.285 |
| 4 |  | -0.029 | -0.031 | -0.030 | -0.139 | -0.082 | -0.084 | -0.080 | -0.275 |
| 6 |  | -0.026 | -0.027 | -0.026 | -0.117 | -0.084 | -0.086 | -0.083 | -0.262 |
| 8 |  | -0.024 | -0.025 | -0.025 | -0.107 | -0.085 | -0.086 | -0.085 | -0.252 |

**Table S4.** Pearson correlation coefficients (*R_p_*) between experimentally determined pKd and the binding affinities calculated by MM/PBSA or MM/GBSA using structures with 5ns MD simulations for the high polarity subset

| εin | Force field | Implicit solvent | | | | Explicit solvent | | | |
| --- | --- | --- | --- | --- | --- | --- | --- | --- | --- |
|  |  | GBHCT | GBOBC1 | GBOBC2 | PBSA | GBHCT | GBOBC1 | GBOBC2 | PBSA |
| 1 | ff14SB | -0.636 | -0.623 | -0.603 | -0.560 | -0.661 | -0.648 | -0.624 | -0.573 |
| 2 |  | -0.604 | -0.593 | -0.585 | -0.580 | -0.634 | -0.626 | -0.620 | -0.654 |
| 4 |  | -0.578 | -0.571 | -0.568 | -0.566 | -0.605 | -0.599 | -0.597 | -0.655 |
| 6 |  | -0.568 | -0.563 | -0.561 | -0.543 | -0.593 | -0.589 | -0.587 | -0.641 |
| 8 |  | -0.563 | -0.559 | -0.557 | -0.525 | -0.587 | -0.583 | -0.582 | -0.629 |
| 1 | ff02 | -0.636 | -0.573 | -0.515 | -0.235 | -0.628 | -0.563 | -0.462 | -0.647 |
| 2 |  | -0.635 | -0.605 | -0.584 | -0.335 | -0.570 | -0.536 | -0.505 | -0.682 |
| 4 |  | -0.624 | -0.608 | -0.599 | -0.362 | -0.531 | -0.512 | -0.500 | -0.637 |
| 6 |  | -0.618 | -0.607 | -0.602 | -0.353 | -0.517 | -0.504 | -0.496 | -0.602 |
| 8 |  | -0.615 | -0.607 | -0.603 | -0.342 | -0.509 | -0.500 | -0.494 | -0.579 |
| 1 | ff03 | -0.678 | -0.655 | -0.650 | -0.609 | -0.768 | -0.754 | -0.750 | -0.573 |
| 2 |  | -0.680 | -0.668 | -0.667 | -0.623 | -0.728 | -0.718 | -0.720 | -0.718 |
| 4 |  | -0.672 | -0.665 | -0.665 | -0.560 | -0.697 | -0.690 | -0.692 | -0.744 |
| 6 |  | -0.668 | -0.663 | -0.663 | -0.518 | -0.685 | -0.680 | -0.681 | -0.738 |
| 8 |  | -0.665 | -0.661 | -0.662 | -0.492 | -0.679 | -0.675 | -0.676 | -0.731 |
| 1 | ff14SBonlysc | -0.723 | -0.713 | -0.704 | -0.616 | -0.646 | -0.623 | -0.595 | -0.570 |
| 2 |  | -0.703 | -0.695 | -0.692 | -0.642 | -0.601 | -0.588 | -0.582 | -0.610 |
| 4 |  | -0.681 | -0.675 | -0.675 | -0.633 | -0.561 | -0.553 | -0.551 | -0.597 |
| 6 |  | -0.671 | -0.667 | -0.667 | -0.617 | -0.545 | -0.539 | -0.538 | -0.576 |
| 8 |  | -0.666 | -0.663 | -0.663 | -0.605 | -0.537 | -0.532 | -0.531 | -0.560 |
| 1 | ff99 | -0.512 | -0.468 | -0.435 | -0.255 | -0.691 | -0.672 | -0.652 | -0.528 |
| 2 |  | -0.559 | -0.542 | -0.527 | -0.371 | -0.666 | -0.654 | -0.650 | -0.664 |
| 4 |  | -0.576 | -0.568 | -0.562 | -0.448 | -0.636 | -0.629 | -0.628 | -0.694 |
| 6 |  | -0.580 | -0.575 | -0.571 | -0.470 | -0.624 | -0.619 | -0.618 | -0.685 |
| 8 |  | -0.582 | -0.578 | -0.575 | -0.478 | -0.618 | -0.613 | -0.613 | -0.676 |
| 1 | rsff2* | -0.483 | -0.470 | -0.453 | -0.187 | -0.631 | -0.583 | -0.535 | -0.555 |
| 2 |  | -0.442 | -0.431 | -0.424 | -0.095 | -0.606 | -0.585 | -0.570 | -0.623 |
| 4 |  | -0.404 | -0.395 | -0.393 | -0.014 | -0.577 | -0.566 | -0.561 | -0.615 |
| 6 |  | -0.388 | -0.381 | -0.379 | 0.019 | -0.565 | -0.558 | -0.554 | -0.593 |
| 8 |  | -0.378 | -0.373 | -0.372 | 0.036 | -0.559 | -0.553 | -0.551 | -0.577 |

**Table S5.** Pearson correlation coefficients (*R_p_*) between experimentally determined pKd and the binding affinities calculated by MM/PBSA or MM/GBSA using structures with 5ns MD simulations for the low polarity subset

| εin | Force field | Implicit solvent | | | | Explicit solvent | | | |
| --- | --- | --- | --- | --- | --- | --- | --- | --- | --- |
|  |  | GBHCT | GBOBC1 | GBOBC2 | PBSA | GBHCT | GBOBC1 | GBOBC2 | PBSA |
| 1 | ff14SB | -0.070 | -0.112 | -0.105 | -0.329 | -0.224 | -0.295 | -0.309 | -0.300 |
| 2 |  | -0.038 | -0.053 | -0.051 | -0.364 | -0.132 | -0.149 | -0.156 | -0.340 |
| 4 |  | -0.022 | -0.027 | -0.026 | -0.342 | -0.093 | -0.098 | -0.099 | -0.306 |
| 6 |  | -0.017 | -0.020 | -0.019 | -0.301 | -0.082 | -0.084 | -0.085 | -0.275 |
| 8 |  | -0.014 | -0.016 | -0.015 | -0.271 | -0.076 | -0.078 | -0.078 | -0.255 |
| 1 | ff02 | -0.045 | -0.071 | -0.066 | -0.161 | -0.194 | -0.259 | -0.268 | -0.399 |
| 2 |  | -0.009 | -0.013 | -0.008 | -0.191 | -0.101 | -0.110 | -0.116 | -0.364 |
| 4 |  | 0.008 | 0.007 | 0.010 | -0.154 | -0.062 | -0.062 | -0.061 | -0.300 |
| 6 |  | 0.013 | 0.013 | 0.014 | -0.129 | -0.050 | -0.050 | -0.049 | -0.263 |
| 8 |  | 0.015 | 0.016 | 0.017 | -0.114 | -0.045 | -0.044 | -0.043 | -0.241 |
| 1 | ff03 | 0.171 | 0.178 | 0.207 | -0.347 | 0.080 | 0.132 | 0.218 | -0.084 |
| 2 |  | 0.126 | 0.123 | 0.137 | -0.419 | 0.011 | 0.020 | 0.049 | -0.158 |
| 4 |  | 0.102 | 0.099 | 0.105 | -0.383 | -0.016 | -0.015 | -0.004 | -0.195 |
| 6 |  | 0.094 | 0.092 | 0.095 | -0.324 | -0.024 | -0.024 | -0.017 | -0.196 |
| 8 |  | 0.090 | 0.088 | 0.091 | -0.286 | -0.028 | -0.028 | -0.023 | -0.195 |
| 1 | ff14SBonlysc | -0.038 | -0.053 | -0.028 | -0.233 | -0.125 | -0.169 | -0.158 | -0.300 |
| 2 |  | -0.040 | -0.048 | -0.039 | -0.290 | -0.072 | -0.086 | -0.087 | -0.298 |
| 4 |  | -0.039 | -0.042 | -0.039 | -0.338 | -0.049 | -0.053 | -0.053 | -0.262 |
| 6 |  | -0.039 | -0.041 | -0.038 | -0.341 | -0.042 | -0.044 | -0.044 | -0.233 |
| 8 |  | -0.038 | -0.040 | -0.038 | -0.331 | -0.038 | -0.040 | -0.040 | -0.214 |
| 1 | ff99 | -0.029 | -0.058 | -0.045 | -0.198 | -0.045 | -0.072 | -0.060 | -0.098 |
| 2 |  | -0.048 | -0.064 | -0.060 | -0.259 | -0.025 | -0.033 | -0.028 | -0.134 |
| 4 |  | -0.056 | -0.064 | -0.063 | -0.299 | -0.016 | -0.019 | -0.017 | -0.146 |
| 6 |  | -0.059 | -0.064 | -0.063 | -0.302 | -0.014 | -0.015 | -0.014 | -0.142 |
| 8 |  | -0.060 | -0.063 | -0.063 | -0.300 | -0.013 | -0.014 | -0.013 | -0.138 |
| 1 | rsff2* | 0.084 | 0.090 | 0.067 | -0.030 | -0.034 | -0.026 | 0.016 | -0.085 |
| 2 |  | 0.052 | 0.051 | 0.043 | -0.036 | -0.032 | -0.030 | -0.015 | -0.122 |
| 4 |  | 0.035 | 0.033 | 0.029 | -0.047 | -0.029 | -0.028 | -0.023 | -0.126 |
| 6 |  | 0.029 | 0.028 | 0.025 | -0.053 | -0.029 | -0.028 | -0.025 | -0.115 |
| 8 |  | 0.026 | 0.025 | 0.023 | -0.057 | -0.028 | -0.028 | -0.025 | -0.107 |

**Table S6.** Pearson correlation coefficients (*R_p_*) between experimentally determined pKd and the binding affinities calculated by MM/PBSA or MM/GBSA using energy minimized structures for the short subset

| εin | Force field | Implicit solvent | | | | Explicit solvent | | | |
| --- | --- | --- | --- | --- | --- | --- | --- | --- | --- |
|  |  | GBHCT | GBOBC1 | GBOBC2 | PBSA | GBHCT | GBOBC1 | GBOBC2 | PBSA |
| 1 | ff14SB | 0.030 | 0.074 | 0.132 | -0.184 | 0.058 | 0.117 | 0.168 | 0.037 |
| 2 |  | -0.077 | -0.064 | -0.032 | -0.216 | -0.058 | -0.033 | 0.002 | -0.052 |
| 4 |  | -0.138 | -0.135 | -0.121 | -0.250 | -0.135 | -0.126 | -0.110 | -0.149 |
| 6 |  | -0.157 | -0.156 | -0.147 | -0.250 | -0.160 | -0.155 | -0.145 | -0.185 |
| 8 |  | -0.167 | -0.166 | -0.160 | -0.262 | -0.172 | -0.169 | -0.162 | -0.201 |
| 1 | ff02 | 0.031 | 0.105 | 0.195 | -0.060 | -0.025 | 0.051 | 0.131 | -0.071 |
| 2 |  | -0.051 | -0.025 | 0.017 | -0.088 | -0.106 | -0.079 | -0.041 | -0.118 |
| 4 |  | -0.095 | -0.086 | -0.070 | -0.128 | -0.151 | -0.141 | -0.127 | -0.160 |
| 6 |  | -0.110 | -0.105 | -0.095 | -0.142 | -0.166 | -0.160 | -0.151 | -0.179 |
| 8 |  | -0.117 | -0.114 | -0.107 | -0.149 | -0.173 | -0.169 | -0.163 | -0.188 |
| 1 | ff03 | -0.011 | 0.051 | 0.126 | -0.400 | 0.037 | 0.110 | 0.184 | -0.238 |
| 2 |  | -0.112 | -0.093 | -0.057 | -0.362 | -0.093 | -0.069 | -0.025 | -0.186 |
| 4 |  | -0.157 | -0.151 | -0.137 | -0.324 | -0.159 | -0.151 | -0.133 | -0.218 |
| 6 |  | -0.170 | -0.167 | -0.158 | -0.308 | -0.178 | -0.173 | -0.163 | -0.217 |
| 8 |  | -0.176 | -0.174 | -0.168 | -0.300 | -0.187 | -0.184 | -0.176 | -0.220 |
| 1 | ff14SBonlysc | 0.033 | 0.078 | 0.132 | -0.153 | 0.075 | 0.129 | 0.175 | -0.028 |
| 2 |  | -0.073 | -0.059 | -0.028 | -0.190 | -0.047 | -0.026 | 0.008 | -0.092 |
| 4 |  | -0.133 | -0.130 | -0.116 | -0.224 | -0.130 | -0.124 | -0.109 | -0.160 |
| 6 |  | -0.152 | -0.151 | -0.143 | -0.236 | -0.158 | -0.155 | -0.145 | -0.188 |
| 8 |  | -0.162 | -0.161 | -0.155 | -0.241 | -0.171 | -0.169 | -0.163 | -0.201 |
| 1 | ff99 | 0.065 | 0.111 | 0.165 | -0.130 | -0.001 | 0.060 | 0.114 | -0.078 |
| 2 |  | -0.050 | -0.036 | -0.005 | -0.163 | -0.100 | -0.074 | -0.040 | -0.130 |
| 4 |  | -0.116 | -0.113 | -0.100 | -0.199 | -0.159 | -0.150 | -0.134 | -0.182 |
| 6 |  | -0.138 | -0.137 | -0.129 | -0.209 | -0.178 | -0.173 | -0.163 | -0.202 |
| 8 |  | -0.149 | -0.148 | -0.142 | -0.215 | -0.187 | -0.183 | -0.177 | -0.211 |
| 1 | rsff2* | 0.163 | 0.216 | 0.268 | 0.207 | 0.012 | 0.066 | 0.114 | 0.037 |
| 2 |  | -0.014 | 0.004 | 0.048 | 0.067 | -0.103 | -0.082 | -0.051 | -0.080 |
| 4 |  | -0.142 | -0.140 | -0.120 | -0.097 | -0.173 | -0.166 | -0.152 | -0.190 |
| 6 |  | -0.184 | -0.184 | -0.172 | -0.167 | -0.195 | -0.191 | -0.183 | -0.232 |
| 8 |  | -0.203 | -0.204 | -0.196 | -0.204 | -0.205 | -0.203 | -0.197 | -0.250 |

**Table S7.** Pearson correlation coefficients (*R_p_*) between experimentally determined pKd and the binding affinities calculated by MM/PBSA or MM/GBSA using energy minimized structures for the long subset (based on 100 bootstrapped resampling)

| εin | Force field | Implicit solvent | | | | Explicit solvent | | | |
| --- | --- | --- | --- | --- | --- | --- | --- | --- | --- |
|  |  | GBHCT | GBOBC1 | GBOBC2 | PBSA | GBHCT | GBOBC1 | GBOBC2 | PBSA |
| 1 | ff14SB | -0.387 ±0.115*^a^* | -0.465 ±0.100 | -0.479 ±0.097 | -0.544 ±0.090 | -0.263 ±0.139 | -0.337 ±0.126 | -0.327 ±0.122 | -0.579 ±0.093 |
| 2 |  | -0.328 ±0.129 | -0.367 ±0.122 | -0.381 ±0.118 | -0.572 ±0.094 | -0.315 ±0.138 | -0.358 ±0.130 | -0.360 ±0.127 | -0.604 ±0.090 |
| 4 |  | -0.275 ±0.138 | -0.291 ±0.135 | -0.298 ±0.133 | -0.570 ±0.107 | -0.330 ±0.138 | -0.350 ±0.134 | -0.352 ±0.132 | -0.597 ±0.097 |
| 6 |  | -0.254 ±0.141 | -0.264 ±0.139 | -0.268 ±0.138 | -0.548 ±0.114 | -0.332 ±0.138 | -0.345 ±0.136 | -0.346 ±0.135 | -0.585 ±0.101 |
| 8 |  | -0.243 ±0.142 | -0.250 ±0.141 | -0.253 ±0.140 | -0.526 ±0.117 | -0.333 ±0.138 | -0.342 ±0.136 | -0.343 ±0.136 | -0.574 ±0.103 |
| 1 | ff02 | -0.384 ±0.122 | -0.452 ±0.107 | -0.454 ±0.104 | -0.522 ±0.098 | -0.342 ±0.127 | -0.405 ±0.112 | -0.385 ±0.100 | -0.578 ±0.096 |
| 2 |  | -0.316 ±0.135 | -0.344 ±0.129 | -0.356 ±0.126 | -0.550 ±0.104 | -0.352 ±0.128 | -0.385 ±0.122 | -0.389 ±0.118 | -0.593 ±0.099 |
| 4 |  | -0.264 ±0.142 | -0.274 ±0.140 | -0.279 ±0.138 | -0.545 ±0.118 | -0.342 ±0.129 | -0.356 ±0.127 | -0.359 ±0.125 | -0.574 ±0.103 |
| 6 |  | -0.245 ±0.144 | -0.250 ±0.143 | -0.253 ±0.142 | -0.519 ±0.124 | -0.337 ±0.130 | -0.345 ±0.128 | -0.347 ±0.128 | -0.556 ±0.104 |
| 8 |  | -0.235 ±0.145 | -0.239 ±0.144 | -0.240 ±0.144 | -0.496 ±0.126 | -0.333 ±0.130 | -0.339 ±0.129 | -0.341 ±0.129 | -0.542 ±0.104 |
| 1 | ff03 | -0.374 ±0.122 | -0.435 ±0.110 | -0.437 ±0.107 | -0.574 ±0.078 | -0.263 ±0.134 | -0.316 ±0.126 | -0.299 ±0.125 | -0.528 ±0.101 |
| 2 |  | -0.312 ±0.136 | -0.337 ±0.132 | -0.343 ±0.130 | -0.587 ±0.085 | -0.290 ±0.131 | -0.319 ±0.126 | -0.313 ±0.125 | -0.535 ±0.099 |
| 4 |  | -0.263 ±0.144 | -0.273 ±0.143 | -0.276 ±0.142 | -0.575 ±0.103 | -0.295 ±0.130 | -0.308 ±0.128 | -0.306 ±0.127 | -0.516 ±0.103 |
| 6 |  | -0.245 ±0.147 | -0.250 ±0.146 | -0.252 ±0.145 | -0.541 ±0.112 | -0.295 ±0.130 | -0.303 ±0.129 | -0.302 ±0.128 | -0.497 ±0.105 |
| 8 |  | -0.236 ±0.148 | -0.239 ±0.148 | -0.241 ±0.147 | -0.514 ±0.115 | -0.295 ±0.130 | -0.301 ±0.129 | -0.300 ±0.129 | -0.485 ±0.105 |

*^a^* Values represent the mean ± standard deviation of Rp obtained from 100 bootstrapped resampling of the long-peptide subset (n = 18 per sample).

**Table S7.** (Continued)

| εin | Force field | Implicit solvent | | | | Explicit solvent | | | |
| --- | --- | --- | --- | --- | --- | --- | --- | --- | --- |
|  |  | GBHCT | GBOBC1 | GBOBC2 | PBSA | GBHCT | GBOBC1 | GBOBC2 | PBSA |
| 1 | ff14SBonlysc | -0.394 ±0.118 | -0.475 ±0.104 | -0.491 ±0.101 | -0.538 ±0.093 | -0.244 ±0.139 | -0.320 ±0.125 | -0.307 ±0.122 | -0.575 ±0.095 |
| 2 |  | -0.329 ±0.132 | -0.369 ±0.125 | -0.385 ±0.121 | -0.563 ±0.098 | -0.300 ±0.136 | -0.345 ±0.129 | -0.344 ±0.126 | -0.589 ±0.093 |
| 4 |  | -0.273 ±0.141 | -0.288 ±0.138 | -0.297 ±0.136 | -0.556 ±0.111 | -0.319 ±0.136 | -0.340 ±0.133 | -0.341 ±0.131 | -0.582 ±0.097 |
| 6 |  | -0.250 ±0.144 | -0.260 ±0.142 | -0.265 ±0.141 | -0.532 ±0.117 | -0.323 ±0.137 | -0.336 ±0.135 | -0.337 ±0.134 | -0.569 ±0.100 |
| 8 |  | -0.239 ±0.145 | -0.245 ±0.144 | -0.249 ±0.143 | -0.509 ±0.119 | -0.324 ±0.137 | -0.333 ±0.135 | -0.334 ±0.135 | -0.559 ±0.102 |
| 1 | ff99 | -0.405 ±0.117 | -0.486 ±0.102 | -0.505 ±0.099 | -0.552 ±0.092 | -0.293 ±0.133 | -0.371 ±0.122 | -0.359 ±0.120 | -0.488 ±0.116 |
| 2 |  | -0.341 ±0.131 | -0.381 ±0.124 | -0.399 ±0.120 | -0.583 ±0.097 | -0.331 ±0.132 | -0.376 ±0.125 | -0.376 ±0.123 | -0.517 ±0.108 |
| 4 |  | -0.285 ±0.140 | -0.301 ±0.137 | -0.310 ±0.135 | -0.580 ±0.114 | -0.336 ±0.133 | -0.356 ±0.130 | -0.357 ±0.129 | -0.530 ±0.107 |
| 6 |  | -0.264 ±0.143 | -0.273 ±0.141 | -0.279 ±0.140 | -0.557 ±0.123 | -0.334 ±0.134 | -0.347 ±0.132 | -0.348 ±0.132 | -0.524 ±0.110 |
| 8 |  | -0.252 ±0.144 | -0.259 ±0.143 | -0.263 ±0.142 | -0.535 ±0.127 | -0.333 ±0.135 | -0.342 ±0.133 | -0.342 ±0.133 | -0.519 ±0.110 |
| 1 | rsff2* | -0.331 ±0.114 | -0.425 ±0.100 | -0.443 ±0.097 | -0.565 ±0.090 | -0.210 ±0.139 | -0.279 ±0.130 | -0.266 ±0.131 | -0.437 ±0.115 |
| 2 |  | -0.304 ±0.123 | -0.354 ±0.116 | -0.374 ±0.113 | -0.551 ±0.093 | -0.295 ±0.131 | -0.339 ±0.124 | -0.339 ±0.122 | -0.514 ±0.102 |
| 4 |  | -0.269 ±0.130 | -0.290 ±0.128 | -0.301 ±0.126 | -0.508 ±0.103 | -0.328 ±0.127 | -0.350 ±0.123 | -0.351 ±0.122 | -0.560 ±0.099 |
| 6 |  | -0.253 ±0.133 | -0.266 ±0.131 | -0.273 ±0.130 | -0.478 ±0.109 | -0.336 ±0.126 | -0.349 ±0.123 | -0.351 ±0.122 | -0.566 ±0.097 |
| 8 |  | -0.245 ±0.134 | -0.254 ±0.133 | -0.259 ±0.132 | -0.458 ±0.112 | -0.339 ±0.125 | -0.349 ±0.123 | -0.350 ±0.123 | -0.565 ±0.096 |

**Table S8.** Pearson correlation coefficients (*R_p_*) between experimentally determined pKd and the binding affinities calculated by MM/PBSA or MM/GBSA using structures with 5ns MD simulations for the short subset

| εin | Force field | Implicit solvent | | | | Explicit solvent | | | |
| --- | --- | --- | --- | --- | --- | --- | --- | --- | --- |
|  |  | GBHCT | GBOBC1 | GBOBC2 | PBSA | GBHCT | GBOBC1 | GBOBC2 | PBSA |
| 1 | ff14SB | 0.210 | 0.230 | 0.258 | 0.079 | 0.057 | 0.124 | 0.207 | 0.222 |
| 2 |  | 0.170 | 0.178 | 0.198 | 0.069 | -0.072 | -0.053 | -0.013 | 0.062 |
| 4 |  | 0.135 | 0.137 | 0.148 | 0.049 | -0.139 | -0.134 | -0.118 | -0.070 |
| 6 |  | 0.122 | 0.122 | 0.130 | 0.039 | -0.160 | -0.158 | -0.148 | -0.122 |
| 8 |  | 0.114 | 0.115 | 0.120 | 0.032 | -0.171 | -0.169 | -0.162 | -0.150 |
| 1 | ff02 | -0.046 | -0.003 | 0.067 | -0.004 | -0.064 | 0.022 | 0.171 | -0.123 |
| 2 |  | -0.115 | -0.104 | -0.074 | -0.066 | -0.129 | -0.104 | -0.055 | -0.132 |
| 4 |  | -0.150 | -0.147 | -0.135 | -0.112 | -0.159 | -0.150 | -0.133 | -0.147 |
| 6 |  | -0.162 | -0.160 | -0.153 | -0.128 | -0.168 | -0.163 | -0.153 | -0.156 |
| 8 |  | -0.167 | -0.166 | -0.161 | -0.139 | -0.172 | -0.169 | -0.162 | -0.162 |
| 1 | ff03 | 0.059 | 0.113 | 0.173 | 0.223 | 0.099 | 0.207 | 0.315 | 0.311 |
| 2 |  | -0.135 | -0.128 | -0.088 | -0.007 | -0.081 | -0.049 | 0.014 | 0.088 |
| 4 |  | -0.232 | -0.232 | -0.217 | -0.230 | -0.174 | -0.165 | -0.141 | -0.072 |
| 6 |  | -0.258 | -0.259 | -0.250 | -0.288 | -0.202 | -0.197 | -0.183 | -0.130 |
| 8 |  | -0.270 | -0.271 | -0.265 | -0.315 | -0.215 | -0.212 | -0.202 | -0.163 |
| 1 | ff14SBonlysc | 0.074 | 0.100 | 0.138 | 0.060 | 0.057 | 0.088 | 0.147 | -0.031 |
| 2 |  | -0.030 | -0.026 | 0.002 | 0.008 | -0.056 | -0.051 | -0.017 | -0.086 |
| 4 |  | -0.107 | -0.109 | -0.094 | -0.067 | -0.124 | -0.125 | -0.110 | -0.142 |
| 6 |  | -0.134 | -0.136 | -0.127 | -0.108 | -0.146 | -0.148 | -0.138 | -0.167 |
| 8 |  | -0.147 | -0.149 | -0.143 | -0.134 | -0.157 | -0.158 | -0.152 | -0.182 |
| 1 | ff99 | 0.247 | 0.290 | 0.331 | 0.199 | 0.141 | 0.210 | 0.290 | 0.308 |
| 2 |  | 0.172 | 0.192 | 0.228 | 0.192 | 0.004 | 0.023 | 0.066 | 0.164 |
| 4 |  | 0.101 | 0.106 | 0.127 | 0.163 | -0.078 | -0.074 | -0.056 | 0.036 |
| 6 |  | 0.071 | 0.073 | 0.087 | 0.140 | -0.105 | -0.103 | -0.092 | -0.018 |
| 8 |  | 0.056 | 0.056 | 0.067 | 0.123 | -0.118 | -0.117 | -0.110 | -0.049 |
| 1 | rsff2* | -0.017 | 0.044 | 0.101 | -0.013 | 0.110 | 0.176 | 0.244 | 0.284 |
| 2 |  | -0.171 | -0.153 | -0.112 | -0.132 | -0.014 | 0.012 | 0.058 | 0.169 |
| 4 |  | -0.264 | -0.262 | -0.245 | -0.270 | -0.094 | -0.086 | -0.065 | 0.046 |
| 6 |  | -0.292 | -0.291 | -0.282 | -0.331 | -0.121 | -0.117 | -0.104 | -0.014 |
| 8 |  | -0.304 | -0.304 | -0.297 | -0.362 | -0.135 | -0.132 | -0.123 | -0.048 |

**Table S9.** Pearson correlation coefficients (*R_p_*) between experimentally determined pKd and the binding affinities calculated by MM/PBSA or MM/GBSA using structures with 5ns MD simulations for the long subset (based on 100 bootstrapped resampling)

| εin | Force field | Implicit solvent | | | | Explicit solvent | | | |
| --- | --- | --- | --- | --- | --- | --- | --- | --- | --- |
|  |  | GBHCT | GBOBC1 | GBOBC2 | PBSA | GBHCT | GBOBC1 | GBOBC2 | PBSA |
| 1 | ff14SB | -0.303 ±0.130*^a^* | -0.361 ±0.118 | -0.355 ±0.114 | -0.570 ±0.078 | -0.385 ±0.121 | -0.478 ±0.110 | -0.500 ±0.106 | -0.580 ±0.081 |
| 2 |  | -0.283 ±0.136 | -0.311 ±0.131 | -0.311 ±0.128 | -0.592 ±0.081 | -0.327 ±0.134 | -0.371 ±0.128 | -0.390 ±0.125 | -0.596 ±0.094 |
| 4 |  | -0.260 ±0.139 | -0.271 ±0.137 | -0.272 ±0.136 | -0.583 ±0.100 | -0.272 ±0.144 | -0.290 ±0.142 | -0.298 ±0.140 | -0.565 ±0.107 |
| 6 |  | -0.250 ±0.140 | -0.256 ±0.139 | -0.256 ±0.138 | -0.563 ±0.110 | -0.251 ±0.148 | -0.261 ±0.147 | -0.267 ±0.146 | -0.534 ±0.112 |
| 8 |  | -0.244 ±0.141 | -0.248 ±0.139 | -0.249 ±0.139 | -0.546 ±0.115 | -0.240 ±0.150 | -0.247 ±0.149 | -0.251 ±0.149 | -0.512 ±0.115 |
| 1 | ff02 | -0.229 ±0.148 | -0.258 ±0.140 | -0.242 ±0.141 | -0.251 ±0.165 | -0.347 ±0.106 | -0.416 ±0.093 | -0.429 ±0.092 | -0.597 ±0.094 |
| 2 |  | -0.201 ±0.152 | -0.210 ±0.148 | -0.205 ±0.148 | -0.348 ±0.128 | -0.241 ±0.119 | -0.265 ±0.115 | -0.280 ±0.112 | -0.584 ±0.096 |
| 4 |  | -0.179 ±0.154 | -0.182 ±0.152 | -0.180 ±0.152 | -0.370 ±0.115 | -0.173 ±0.128 | -0.179 ±0.127 | -0.185 ±0.126 | -0.527 ±0.101 |
| 6 |  | -0.172 ±0.154 | -0.173 ±0.153 | -0.171 ±0.153 | -0.358 ±0.116 | -0.149 ±0.131 | -0.152 ±0.131 | -0.155 ±0.130 | -0.480 ±0.102 |
| 8 |  | -0.167 ±0.154 | -0.168 ±0.153 | -0.167 ±0.153 | -0.347 ±0.117 | -0.138 ±0.133 | -0.139 ±0.132 | -0.141 ±0.132 | -0.447 ±0.102 |
| 1 | ff03 | -0.147 ±0.142 | -0.193 ±0.138 | -0.180 ±0.139 | -0.617 ±0.087 | -0.263 ±0.157 | -0.330 ±0.145 | -0.338 ±0.144 | -0.529 ±0.110 |
| 2 |  | -0.161 ±0.141 | -0.185 ±0.141 | -0.179 ±0.141 | -0.630 ±0.068 | -0.249 ±0.153 | -0.281 ±0.148 | -0.289 ±0.148 | -0.568 ±0.099 |
| 4 |  | -0.166 ±0.142 | -0.177 ±0.142 | -0.174 ±0.142 | -0.573 ±0.081 | -0.231 ±0.151 | -0.245 ±0.149 | -0.249 ±0.149 | -0.572 ±0.098 |
| 6 |  | -0.167 ±0.142 | -0.174 ±0.142 | -0.172 ±0.142 | -0.535 ±0.094 | -0.224 ±0.151 | -0.232 ±0.149 | -0.235 ±0.149 | -0.559 ±0.099 |
| 8 |  | -0.167 ±0.142 | -0.172 ±0.142 | -0.171 ±0.142 | -0.511 ±0.102 | -0.220 ±0.150 | -0.226 ±0.149 | -0.228 ±0.149 | -0.548 ±0.099 |

*^a^* Values represent the mean ± standard deviation of Rp obtained from 100 bootstrapped resampling of the long-peptide subset (n = 18 per sample).

**Table S9.** (Continued)

| εin | Force field | Implicit solvent | | | | Explicit solvent | | | |
| --- | --- | --- | --- | --- | --- | --- | --- | --- | --- |
|  |  | GBHCT | GBOBC1 | GBOBC2 | PBSA | GBHCT | GBOBC1 | GBOBC2 | PBSA |
| 1 | ff14SBonlysc | -0.312 ±0.149 | -0.362 ±0.140 | -0.359 ±0.141 | -0.630 ±0.067 | -0.299 ±0.110 | -0.387 ±0.096 | -0.412 ±0.092 | -0.479 ±0.099 |
| 2 |  | -0.292 ±0.155 | -0.316 ±0.150 | -0.318 ±0.150 | -0.652 ±0.075 | -0.213 ±0.118 | -0.253 ±0.113 | -0.273 ±0.110 | -0.448 ±0.103 |
| 4 |  | -0.270 ±0.158 | -0.280 ±0.156 | -0.282 ±0.156 | -0.640 ±0.091 | -0.149 ±0.122 | -0.164 ±0.120 | -0.174 ±0.119 | -0.395 ±0.107 |
| 6 |  | -0.261 ±0.159 | -0.267 ±0.158 | -0.268 ±0.158 | -0.623 ±0.098 | -0.125 ±0.124 | -0.134 ±0.123 | -0.140 ±0.122 | -0.357 ±0.109 |
| 8 |  | -0.256 ±0.160 | -0.260 ±0.159 | -0.260 ±0.159 | -0.609 ±0.102 | -0.113 ±0.125 | -0.119 ±0.124 | -0.123 ±0.124 | -0.331 ±0.109 |
| 1 | ff99 | -0.208 ±0.143 | -0.239 ±0.135 | -0.224 ±0.135 | -0.281 ±0.138 | -0.341 ±0.138 | -0.412 ±0.126 | -0.436 ±0.120 | -0.468 ±0.115 |
| 2 |  | -0.255 ±0.154 | -0.276 ±0.150 | -0.270 ±0.149 | -0.388 ±0.113 | -0.290 ±0.147 | -0.322 ±0.142 | -0.339 ±0.140 | -0.524 ±0.118 |
| 4 |  | -0.270 ±0.160 | -0.281 ±0.159 | -0.279 ±0.158 | -0.458 ±0.112 | -0.244 ±0.150 | -0.256 ±0.148 | -0.264 ±0.147 | -0.529 ±0.130 |
| 6 |  | -0.274 ±0.162 | -0.280 ±0.161 | -0.279 ±0.161 | -0.476 ±0.118 | -0.226 ±0.151 | -0.233 ±0.150 | -0.238 ±0.149 | -0.509 ±0.132 |
| 8 |  | -0.275 ±0.163 | -0.279 ±0.162 | -0.279 ±0.162 | -0.481 ±0.121 | -0.216 ±0.152 | -0.221 ±0.151 | -0.225 ±0.150 | -0.493 ±0.131 |
| 1 | rsff2* | 0.124 ±0.146 | 0.058 ±0.144 | 0.008 ±0.142 | -0.203 ±0.141 | -0.241 ±0.118 | -0.301 ±0.107 | -0.302 ±0.106 | -0.483 ±0.091 |
| 2 |  | 0.143 ±0.135 | 0.110 ±0.132 | 0.088 ±0.130 | -0.117 ±0.129 | -0.192 ±0.128 | -0.220 ±0.124 | -0.227 ±0.122 | -0.471 ±0.098 |
| 4 |  | 0.150 ±0.131 | 0.133 ±0.130 | 0.124 ±0.127 | -0.038 ±0.121 | -0.151 ±0.136 | -0.162 ±0.134 | -0.165 ±0.133 | -0.406 ±0.106 |
| 6 |  | 0.152 ±0.133 | 0.140 ±0.130 | 0.135 ±0.128 | -0.006 ±0.121 | -0.135 ±0.139 | -0.141 ±0.138 | -0.143 ±0.137 | -0.358 ±0.110 |
| 8 |  | 0.151 ±0.128 | 0.143 ±0.130 | 0.140 ±0.129 | 0.011 ±0.121 | -0.127 ±0.140 | -0.131 ±0.140 | -0.132 ±0.139 | -0.327 ±0.112 |

**Table S10.** The ranks given by ADCP, Rosetta and MM/GB(PB)SA using ff03 force field and implicit solvent model for dataset *II*

| PDB code | Peptide length | Polar interface (%) | ADCP | Rosetta | ff03 implicit (ε_in_) | | | | | | | | | | | |
| --- | --- | --- | --- | --- | --- | --- | --- | --- | --- | --- | --- | --- | --- | --- | --- | --- |
|  |  |  |  |  | GBSA^HCT^ | | | GBSA^OBC1^ | | | GBSA^OBC2^ | | | PBSA | | |
|  |  |  |  |  | 1 | 2 | 4 | 1 | 2 | 4 | 1 | 2 | 4 | 1 | 2 | 4 |
| 1HQQ | 13 | 64.54 | 5*^a^* | 41 | 7 | 12 | 20 | 6 | 12 | 19 | 6 | 11 | 19 | 14 | 16 | 18 |
| 1HXL | 13 | 57.97 | 69 | 73 | 23 | 13 | 9 | 24 | 13 | 9 | 25 | 12 | 9 | 27 | 24 | 25 |
| 1HXZ | 13 | 59.95 | 10 | 32 | 1 | 1 | 2 | 1 | 1 | 2 | 1 | 1 | 1 | 9 | 5 | 2 |
| 1JK4 | 6 | 61.81 | 2 | 1 | 7 | 9 | 9 | 6 | 8 | 9 | 5 | 7 | 9 | 11 | 16 | 10 |
| 1SFI | 14 | 61.97 | 15 | 10 | 26 | 32 | 40 | 28 | 36 | 42 | 28 | 38 | 43 | 5 | 8 | 12 |
| 1SLD | 6 | 73.42 | 10 | 19 | 36 | 32 | 20 | 34 | 28 | 19 | 33 | 28 | 19 | 25 | 17 | 17 |
| 1SLE | 8 | 77.4 | 7 | 17 | 41 | 21 | 17 | 41 | 23 | 17 | 43 | 26 | 18 | 12 | 11 | 12 |
| 1SMF | 9 | 66.97 | 1 | 5 | 1 | 1 | 1 | 1 | 1 | 1 | 1 | 1 | 1 | 1 | 1 | 1 |
| 1VWB | 6 | 73.17 | 5 | 3 | 8 | 3 | 2 | 6 | 3 | 2 | 8 | 4 | 2 | 10 | 12 | 6 |
| 1VWC | 6 | 69.6 | 3 | 17 | 9 | 4 | 4 | 7 | 4 | 3 | 11 | 4 | 3 | 3 | 3 | 2 |
| 1VWD | 6 | 71.95 | 5 | 6 | 8 | 4 | 3 | 10 | 4 | 2 | 13 | 6 | 3 | 14 | 14 | 17 |
| 1VWE | 6 | 73.67 | 23 | 11 | 4 | 8 | 18 | 4 | 9 | 18 | 3 | 8 | 15 | 2 | 3 | 5 |
| 1VWM | 6 | 73.47 | 73 | 80 | 54 | 40 | 33 | 65 | 43 | 35 | 68 | 54 | 41 | 72 | 70 | 65 |
| 1VWN | 6 | 73.35 | 60 | 25 | 11 | 9 | 14 | 14 | 11 | 15 | 17 | 11 | 15 | 12 | 10 | 7 |
| 1YF4 | 9 | 50.68 | 1 | 16 | 1 | 1 | 2 | 1 | 1 | 2 | 1 | 1 | 2 | 1 | 1 | 1 |
| 2BR8 | 16 | 67.27 | 8 | 78 | 8 | 7 | 9 | 4 | 4 | 6 | 3 | 4 | 6 | 12 | 2 | 1 |
| 2C9T | 12 | 58.25 | 1 | 12 | 1 | 1 | 2 | 1 | 1 | 2 | 1 | 1 | 2 | 1 | 2 | 1 |
| 2NWN | 12 | 51.01 | 28 | 66 | 1 | 1 | 1 | 2 | 1 | 1 | 2 | 1 | 1 | 3 | 3 | 3 |
| 2UZ6 | 16 | 66.76 | 2 | 1 | 2 | 2 | 1 | 2 | 1 | 1 | 2 | 1 | 1 | 1 | 2 | 2 |
| 3AV9 | 8 | 61.29 | 7 | 1 | 1 | 8 | 7 | 2 | 8 | 7 | 2 | 7 | 6 | 2 | 2 | 1 |
| 3AVA | 8 | 61.58 | 9 | 4 | 2 | 5 | 9 | 5 | 5 | 10 | 7 | 6 | 11 | 4 | 3 | 2 |
| 3AVB | 8 | 61.38 | 2 | 2 | 1 | 1 | 2 | 1 | 1 | 2 | 1 | 1 | 2 | 10 | 7 | 6 |
| 3AVC | 8 | 62.3 | 33 | 3 | 3 | 1 | 1 | 7 | 1 | 1 | 10 | 1 | 1 | 16 | 16 | 7 |
| 3AVF | 8 | 59.7 | 14 | 1 | 1 | 2 | 3 | 1 | 1 | 3 | 1 | 1 | 2 | 1 | 1 | 4 |
| 3AVG | 8 | 61.83 | 4 | 3 | 1 | 1 | 4 | 1 | 1 | 4 | 1 | 1 | 3 | 1 | 2 | 2 |
| 3AVH | 8 | 62.45 | 2 | 1 | 4 | 2 | 4 | 3 | 2 | 4 | 3 | 2 | 4 | 3 | 3 | 7 |
| 3AVI | 8 | 63.35 | 2 | 5 | 1 | 3 | 5 | 1 | 3 | 4 | 1 | 2 | 4 | 5 | 1 | 1 |
| 3AVJ | 8 | 64.8 | 3 | 6 | 1 | 1 | 1 | 1 | 1 | 1 | 1 | 1 | 1 | 1 | 1 | 2 |
| 3AVK | 8 | 63.12 | 1 | 1 | 1 | 1 | 1 | 1 | 1 | 1 | 1 | 1 | 1 | 1 | 1 | 1 |
| 3AVL | 8 | 61.54 | 11 | 1 | 1 | 2 | 3 | 1 | 2 | 3 | 1 | 2 | 3 | 1 | 2 | 4 |
| 3AVM | 8 | 61.26 | 2 | 1 | 2 | 1 | 1 | 4 | 1 | 1 | 6 | 1 | 1 | 3 | 4 | 2 |
| 3AVN | 8 | 61.76 | 14 | 1 | 1 | 1 | 5 | 1 | 1 | 5 | 1 | 1 | 5 | 2 | 1 | 2 |
| 3M61 | 12 | 49.48 | 17 | 12 | 1 | 1 | 3 | 1 | 1 | 3 | 2 | 2 | 3 | 13 | 4 | 2 |
| 3P72 | 11 | 62.62 | 3 | 7 | 4 | 30 | 44 | 4 | 30 | 46 | 4 | 27 | 44 | 4 | 7 | 18 |
| 3P8F | 14 | 65.48 | 3 | 28 | 2 | 2 | 2 | 2 | 2 | 2 | 2 | 2 | 2 | 2 | 2 | 2 |
| 3WNE | 6 | 63.78 | 1 | 1 | 1 | 1 | 1 | 1 | 1 | 1 | 1 | 1 | 1 | 1 | 1 | 1 |
| 3WNF | 6 | 65.77 | 4 | 10 | 14 | 13 | 9 | 13 | 13 | 8 | 12 | 11 | 8 | 11 | 10 | 8 |
| 3ZGC | 7 | 48.78 | 2 | 11 | 2 | 5 | 9 | 2 | 5 | 8 | 2 | 5 | 8 | 8 | 6 | 7 |
| 4EZ1 | 13 | 71.56 | 1 | 7 | 32 | 43 | 43 | 42 | 45 | 47 | 41 | 46 | 46 | 83 | 90 | 85 |
| 4GLY | 13 | 48.12 | 3 | 12 | 4 | 8 | 9 | 4 | 7 | 9 | 4 | 7 | 9 | 1 | 1 | 1 |
| 4K1E | 14 | 65.55 | 19 | 60 | 1 | 4 | 10 | 2 | 6 | 11 | 6 | 8 | 12 | 2 | 1 | 1 |
| 4KEL | 14 | 65.36 | 16 | 19 | 6 | 6 | 6 | 6 | 6 | 6 | 5 | 6 | 6 | 2 | 2 | 3 |
| 4OU3 | 6 | 42.09 | 7 | 19 | 59 | 43 | 18 | 59 | 42 | 20 | 59 | 48 | 22 | 83 | 81 | 66 |
| 4X1Q | 10 | 56.36 | 3 | 26 | 1 | 1 | 1 | 1 | 1 | 1 | 1 | 1 | 1 | 1 | 1 | 1 |
| 4XOJ | 13 | 60.96 | 3 | 7 | 1 | 1 | 1 | 1 | 1 | 1 | 1 | 1 | 1 | 1 | 1 | 1 |
| 4Z09 | 13 | 65.07 | 3 | 9 | 1 | 3 | 6 | 1 | 3 | 7 | 1 | 4 | 7 | 1 | 1 | 3 |
| 4ZHL | 10 | 59.9 | 6 | 10 | 1 | 1 | 1 | 1 | 1 | 1 | 1 | 1 | 1 | 1 | 1 | 1 |
| 4ZKS | 12 | 49.14 | 49 | 78 | 14 | 6 | 6 | 18 | 7 | 6 | 22 | 11 | 7 | 32 | 28 | 20 |
| 5CO5 | 16 | 62.1 | 2 | 15 | 7 | 14 | 18 | 7 | 12 | 18 | 6 | 12 | 16 | 1 | 11 | 21 |
| 5DI8 | 13 | 65.33 | 2 | 8 | 7 | 4 | 4 | 4 | 2 | 4 | 7 | 3 | 4 | 11 | 4 | 2 |
| 5DJ0 | 13 | 65.82 | 67 | 11 | 83 | 79 | 75 | 81 | 77 | 76 | 86 | 81 | 77 | 30 | 32 | 47 |
| 5DJ6 | 13 | 65 | 5 | 15 | 33 | 28 | 29 | 28 | 23 | 27 | 30 | 25 | 27 | 2 | 2 | 1 |
| 5DJ8 | 13 | 66.31 | 24 | 15 | 5 | 6 | 9 | 5 | 5 | 9 | 4 | 5 | 9 | 17 | 12 | 9 |
| 5DJC | 13 | 63.72 | 4 | 18 | 5 | 5 | 4 | 4 | 5 | 4 | 4 | 5 | 4 | 3 | 3 | 3 |
| 5DJX | 13 | 66.91 | 25 | 2 | 36 | 60 | 72 | 32 | 58 | 71 | 31 | 60 | 71 | 29 | 38 | 54 |
| 5DJY | 13 | 63.52 | 26 | 14 | 24 | 22 | 21 | 20 | 18 | 18 | 21 | 18 | 18 | 2 | 5 | 11 |
| 5DJZ | 13 | 64.62 | 2 | 27 | 9 | 7 | 6 | 10 | 6 | 6 | 10 | 6 | 6 | 5 | 4 | 4 |
| 5DK0 | 13 | 66.71 | 1 | 49 | 1 | 1 | 1 | 1 | 1 | 1 | 1 | 1 | 1 | 4 | 1 | 1 |
| 5DVL | 13 | 69.61 | 2 | 37 | 6 | 7 | 7 | 5 | 5 | 7 | 6 | 4 | 7 | 14 | 1 | 1 |
| 5DVN | 13 | 70.37 | 42 | 9 | 7 | 10 | 15 | 12 | 13 | 15 | 14 | 12 | 15 | 38 | 45 | 62 |
| 5EOC | 13 | 61.52 | 49 | 46 | 96 | 95 | 94 | 95 | 95 | 94 | 92 | 94 | 93 | 20 | 38 | 60 |
| 5GRD | 10 | 59.7 | 2 | 1 | 1 | 1 | 1 | 1 | 1 | 1 | 1 | 1 | 1 | 2 | 3 | 1 |
| 5GRG | 4 | 54.93 | 1 | 1 | 1 | 1 | 1 | 1 | 1 | 1 | 1 | 1 | 1 | 1 | 1 | 1 |
| 5JZU | 13 | 57.73 | 9 | 6 | 4 | 7 | 9 | 3 | 6 | 9 | 3 | 6 | 9 | 3 | 1 | 3 |
| 5TU6 | 7 | 69.69 | 80 | 91 | 63 | 55 | 50 | 63 | 54 | 50 | 68 | 61 | 52 | 62 | 53 | 44 |
| 5XN3 | 8 | 49.33 | 5 | 1 | 1 | 8 | 12 | 1 | 9 | 13 | 1 | 8 | 12 | 1 | 2 | 9 |
| 6BVH | 14 | 58.18 | 64 | 30 | 8 | 14 | 20 | 9 | 14 | 20 | 9 | 14 | 20 | 4 | 6 | 6 |
| 6D3Y | 14 | 58.28 | 2 | 25 | 1 | 1 | 1 | 1 | 1 | 1 | 1 | 1 | 1 | 1 | 1 | 1 |
| 6D3Z | 14 | 62.3 | 2 | 28 | 1 | 1 | 2 | 1 | 1 | 2 | 1 | 1 | 2 | 1 | 1 | 2 |
| 6XVD | 12 | 49.76 | 3 | 17 | 1 | 1 | 1 | 1 | 1 | 1 | 1 | 1 | 1 | 1 | 1 | 1 |
| 7BB6 | 9 | 70.79 | 46 | 9 | 40 | 50 | 59 | 38 | 52 | 58 | 33 | 41 | 58 | 24 | 36 | 45 |
| 7BB7 | 9 | 70.55 | 16 | 8 | 39 | 44 | 45 | 41 | 43 | 46 | 38 | 43 | 45 | 30 | 39 | 50 |
| 7DW9 | 9 | 61.83 | 2 | 1 | 1 | 1 | 1 | 1 | 1 | 1 | 1 | 1 | 1 | 1 | 1 | 1 |
| 7K2E | 7 | 48.6 | 1 | 14 | 10 | 10 | 16 | 9 | 10 | 15 | 9 | 10 | 12 | 8 | 6 | 7 |
| 7K2F | 7 | 48.76 | 5 | 1 | 1 | 1 | 3 | 1 | 1 | 3 | 1 | 1 | 3 | 2 | 3 | 4 |
| 7K2G | 7 | 47.51 | 13 | 8 | 1 | 6 | 7 | 1 | 5 | 7 | 1 | 5 | 7 | 19 | 11 | 9 |
| 7K2H | 7 | 50.96 | 1 | 1 | 18 | 16 | 7 | 19 | 19 | 7 | 18 | 18 | 8 | 24 | 21 | 20 |
| 7K2I | 7 | 52.4 | 3 | 1 | 1 | 1 | 1 | 1 | 1 | 1 | 1 | 1 | 1 | 3 | 4 | 2 |
| 7K2M | 7 | 50.72 | 1 | 1 | 1 | 2 | 3 | 1 | 2 | 3 | 1 | 1 | 3 | 1 | 1 | 2 |
| 7N0W | 14 | 62.2 | 1 | 8 | 1 | 2 | 1 | 1 | 1 | 1 | 1 | 1 | 1 | 1 | 1 | 2 |
| 7N43 | 17 | 72.08 | 2 | 18 | 53 | 39 | 31 | 55 | 38 | 30 | 60 | 37 | 30 | 9 | 20 | 37 |

*^a^* The order of the first conformation that meets the success criteria

**Table S11.** The ranks given by ADCP, Rosetta and MM/GB(PB)SA using ff03 force field and explicit solvent model for for dataset *II*

| PDB code | Peptide length | Polar interface (%) | ADCP | Rosetta | ff03 explicit (ε_in_) | | | | | | | | | | | |
| --- | --- | --- | --- | --- | --- | --- | --- | --- | --- | --- | --- | --- | --- | --- | --- | --- |
|  |  |  |  |  | GBSA^HCT^ | | | GBSA^OBC1^ | | | GBSA^OBC2^ | | | PBSA | | |
|  |  |  |  |  | 1 | 2 | 4 | 1 | 2 | 4 | 1 | 2 | 4 | 1 | 2 | 4 |
| 1HQQ | 13 | 64.54 | 5*^a^* | 41 | 21 | 11 | 11 | 15 | 10 | 9 | 13 | 7 | 8 | 29 | 12 | 4 |
| 1HXL | 13 | 57.97 | 69 | 73 | 52 | 47 | 46 | 49 | 44 | 46 | 47 | 43 | 45 | 41 | 51 | 65 |
| 1HXZ | 13 | 59.95 | 10 | 32 | 2 | 2 | 4 | 3 | 3 | 5 | 3 | 2 | 4 | 18 | 5 | 5 |
| 1JK4 | 6 | 61.81 | 2 | 1 | 3 | 13 | 15 | 2 | 13 | 16 | 2 | 9 | 16 | 4 | 12 | 14 |
| 1SFI | 14 | 61.97 | 15 | 10 | 41 | 47 | 48 | 45 | 48 | 50 | 52 | 53 | 50 | 12 | 15 | 24 |
| 1SLD | 6 | 73.42 | 10 | 19 | 53 | 37 | 19 | 55 | 39 | 20 | 57 | 44 | 20 | 24 | 43 | 33 |
| 1SLE | 8 | 77.4 | 7 | 17 | 25 | 25 | 20 | 27 | 26 | 21 | 30 | 27 | 21 | 16 | 27 | 25 |
| 1SMF | 9 | 66.97 | 1 | 5 | 1 | 1 | 1 | 1 | 1 | 1 | 2 | 1 | 1 | 1 | 1 | 1 |
| 1VWB | 6 | 73.17 | 5 | 3 | 3 | 4 | 3 | 4 | 4 | 3 | 5 | 4 | 3 | 3 | 5 | 5 |
| 1VWC | 6 | 69.6 | 3 | 17 | 30 | 12 | 8 | 33 | 14 | 9 | 38 | 21 | 12 | 30 | 14 | 11 |
| 1VWD | 6 | 71.95 | 5 | 6 | 7 | 11 | 11 | 12 | 10 | 10 | 14 | 11 | 10 | 13 | 10 | 13 |
| 1VWE | 6 | 73.67 | 23 | 11 | 5 | 12 | 18 | 3 | 10 | 18 | 3 | 9 | 18 | 2 | 3 | 4 |
| 1VWM | 6 | 73.47 | 73 | 80 | 10 | 21 | 27 | 17 | 26 | 30 | 24 | 28 | 31 | 21 | 33 | 44 |
| 1VWN | 6 | 73.35 | 60 | 25 | 4 | 14 | 32 | 4 | 15 | 35 | 5 | 16 | 35 | 1 | 2 | 5 |
| 1YF4 | 9 | 50.68 | 1 | 16 | 1 | 1 | 1 | 1 | 1 | 1 | 1 | 1 | 1 | 1 | 1 | 1 |
| 2BR8 | 16 | 67.27 | 8 | 78 | 15 | 8 | 8 | 5 | 7 | 7 | 4 | 7 | 7 | 35 | 11 | 5 |
| 2C9T | 12 | 58.25 | 1 | 12 | 2 | 2 | 2 | 2 | 3 | 3 | 2 | 3 | 3 | 1 | 2 | 2 |
| 2NWN | 12 | 51.01 | 28 | 66 | 1 | 3 | 6 | 4 | 3 | 6 | 4 | 4 | 6 | 14 | 7 | 4 |
| 2UZ6 | 16 | 66.76 | 2 | 1 | 1 | 1 | 1 | 1 | 1 | 1 | 1 | 1 | 1 | 2 | 2 | 1 |
| 3AV9 | 8 | 61.29 | 7 | 1 | 12 | 11 | 6 | 13 | 10 | 6 | 13 | 12 | 6 | 12 | 21 | 10 |
| 3AVA | 8 | 61.58 | 9 | 4 | 29 | 21 | 18 | 39 | 23 | 18 | 35 | 27 | 18 | 4 | 10 | 20 |
| 3AVB | 8 | 61.38 | 2 | 2 | 2 | 3 | 6 | 2 | 3 | 6 | 2 | 3 | 5 | 2 | 3 | 4 |
| 3AVC | 8 | 62.3 | 33 | 3 | 18 | 4 | 1 | 28 | 6 | 1 | 31 | 9 | 2 | 6 | 11 | 6 |
| 3AVF | 8 | 59.7 | 14 | 1 | 2 | 5 | 3 | 3 | 5 | 3 | 3 | 5 | 3 | 1 | 4 | 8 |
| 3AVG | 8 | 61.83 | 4 | 3 | 3 | 9 | 11 | 2 | 9 | 11 | 2 | 8 | 10 | 1 | 5 | 10 |
| 3AVH | 8 | 62.45 | 2 | 1 | 3 | 6 | 3 | 3 | 5 | 3 | 3 | 5 | 3 | 1 | 2 | 13 |
| 3AVI | 8 | 63.35 | 2 | 5 | 1 | 1 | 1 | 1 | 1 | 1 | 1 | 1 | 1 | 1 | 1 | 1 |
| 3AVJ | 8 | 64.8 | 3 | 6 | 1 | 2 | 2 | 1 | 2 | 2 | 1 | 2 | 2 | 1 | 1 | 2 |
| 3AVK | 8 | 63.12 | 1 | 1 | 1 | 1 | 1 | 1 | 1 | 1 | 1 | 1 | 1 | 1 | 1 | 1 |
| 3AVL | 8 | 61.54 | 11 | 1 | 11 | 10 | 5 | 12 | 9 | 6 | 12 | 12 | 7 | 2 | 4 | 8 |
| 3AVM | 8 | 61.26 | 2 | 1 | 6 | 2 | 1 | 14 | 2 | 1 | 19 | 5 | 2 | 5 | 1 | 1 |
| 3AVN | 8 | 61.76 | 14 | 1 | 8 | 11 | 9 | 9 | 12 | 9 | 10 | 14 | 10 | 20 | 17 | 15 |
| 3M61 | 12 | 49.48 | 17 | 12 | 1 | 2 | 7 | 2 | 4 | 10 | 2 | 5 | 11 | 5 | 6 | 9 |
| 3P72 | 11 | 62.62 | 3 | 7 | 12 | 49 | 61 | 11 | 42 | 57 | 10 | 35 | 54 | 3 | 10 | 27 |
| 3P8F | 14 | 65.48 | 3 | 28 | 1 | 2 | 2 | 1 | 2 | 2 | 1 | 2 | 2 | 1 | 1 | 1 |
| 3WNE | 6 | 63.78 | 1 | 1 | 1 | 1 | 1 | 1 | 1 | 1 | 1 | 1 | 1 | 1 | 1 | 1 |
| 3WNF | 6 | 65.77 | 4 | 10 | 12 | 9 | 9 | 11 | 11 | 9 | 13 | 11 | 13 | 4 | 1 | 1 |
| 3ZGC | 7 | 48.78 | 2 | 11 | 4 | 7 | 28 | 2 | 7 | 29 | 2 | 4 | 27 | 9 | 12 | 19 |
| 4EZ1 | 13 | 71.56 | 1 | 7 | 80 | 78 | 75 | 71 | 76 | 74 | 61 | 70 | 71 | 82 | 88 | 86 |
| 4GLY | 13 | 48.12 | 3 | 12 | 3 | 7 | 14 | 4 | 8 | 15 | 5 | 8 | 16 | 2 | 2 | 4 |
| 4K1E | 14 | 65.55 | 19 | 60 | 2 | 5 | 7 | 4 | 5 | 7 | 4 | 5 | 7 | 1 | 1 | 1 |
| 4KEL | 14 | 65.36 | 16 | 19 | 6 | 8 | 9 | 6 | 9 | 9 | 9 | 9 | 10 | 10 | 7 | 3 |
| 4OU3 | 6 | 42.09 | 7 | 19 | 60 | 27 | 13 | 64 | 25 | 13 | 59 | 26 | 11 | 67 | 65 | 36 |
| 4X1Q | 10 | 56.36 | 3 | 26 | 1 | 1 | 1 | 1 | 1 | 1 | 1 | 1 | 1 | 1 | 1 | 1 |
| 4XOJ | 13 | 60.96 | 3 | 7 | 1 | 1 | 1 | 1 | 1 | 1 | 2 | 1 | 1 | 1 | 1 | 1 |
| 4Z09 | 13 | 65.07 | 3 | 9 | 3 | 7 | 7 | 3 | 6 | 7 | 3 | 7 | 7 | 1 | 4 | 5 |
| 4ZHL | 10 | 59.9 | 6 | 10 | 1 | 2 | 3 | 1 | 2 | 3 | 2 | 2 | 3 | 1 | 1 | 1 |
| 4ZKS | 12 | 49.14 | 49 | 78 | 8 | 9 | 12 | 2 | 8 | 14 | 2 | 8 | 13 | 11 | 5 | 7 |
| 5CO5 | 16 | 62.1 | 2 | 15 | 16 | 19 | 22 | 15 | 18 | 22 | 13 | 17 | 22 | 1 | 5 | 12 |
| 5DI8 | 13 | 65.33 | 2 | 8 | 4 | 4 | 5 | 3 | 5 | 5 | 4 | 5 | 5 | 1 | 1 | 1 |
| 5DJ0 | 13 | 65.82 | 67 | 11 | 19 | 23 | 25 | 18 | 21 | 25 | 18 | 19 | 25 | 14 | 9 | 7 |
| 5DJ6 | 13 | 65 | 5 | 15 | 20 | 14 | 14 | 19 | 14 | 14 | 25 | 17 | 14 | 1 | 2 | 4 |
| 5DJ8 | 13 | 66.31 | 24 | 15 | 16 | 12 | 11 | 12 | 12 | 13 | 10 | 10 | 11 | 25 | 12 | 7 |
| 5DJC | 13 | 63.72 | 4 | 18 | 2 | 4 | 3 | 2 | 4 | 3 | 2 | 4 | 3 | 3 | 4 | 8 |
| 5DJX | 13 | 66.91 | 25 | 2 | 63 | 76 | 85 | 60 | 78 | 86 | 60 | 74 | 85 | 49 | 63 | 83 |
| 5DJY | 13 | 63.52 | 26 | 14 | 24 | 25 | 27 | 24 | 26 | 27 | 24 | 26 | 28 | 9 | 11 | 18 |
| 5DJZ | 13 | 64.62 | 2 | 27 | 2 | 2 | 3 | 3 | 2 | 3 | 3 | 2 | 3 | 1 | 1 | 1 |
| 5DK0 | 13 | 66.71 | 1 | 49 | 2 | 1 | 1 | 2 | 1 | 1 | 2 | 1 | 1 | 7 | 5 | 3 |
| 5DVL | 13 | 69.61 | 2 | 37 | 12 | 9 | 11 | 10 | 9 | 10 | 11 | 9 | 10 | 6 | 2 | 2 |
| 5DVN | 13 | 70.37 | 42 | 9 | 4 | 4 | 10 | 5 | 6 | 11 | 6 | 5 | 9 | 29 | 32 | 37 |
| 5EOC | 13 | 61.52 | 49 | 46 | 72 | 80 | 84 | 73 | 81 | 84 | 73 | 80 | 84 | 15 | 24 | 36 |
| 5GRD | 10 | 59.7 | 2 | 1 | 1 | 1 | 1 | 1 | 1 | 1 | 1 | 1 | 1 | 1 | 1 | 1 |
| 5GRG | 4 | 54.93 | 1 | 1 | 1 | 1 | 1 | 1 | 1 | 1 | 1 | 1 | 1 | 1 | 1 | 1 |
| 5JZU | 13 | 57.73 | 9 | 6 | 4 | 8 | 8 | 4 | 8 | 8 | 5 | 7 | 8 | 5 | 1 | 2 |
| 5TU6 | 7 | 69.69 | 80 | 91 | 45 | 34 | 26 | 48 | 35 | 29 | 56 | 39 | 30 | 80 | 66 | 54 |
| 5XN3 | 8 | 49.33 | 5 | 1 | 1 | 2 | 9 | 1 | 1 | 10 | 1 | 1 | 9 | 1 | 1 | 4 |
| 6BVH | 14 | 58.18 | 64 | 30 | 7 | 10 | 13 | 4 | 12 | 14 | 6 | 12 | 14 | 1 | 1 | 2 |
| 6D3Y | 14 | 58.28 | 2 | 25 | 1 | 2 | 4 | 1 | 3 | 4 | 1 | 3 | 4 | 1 | 1 | 1 |
| 6D3Z | 14 | 62.3 | 2 | 28 | 1 | 5 | 8 | 1 | 7 | 9 | 1 | 7 | 9 | 1 | 1 | 2 |
| 6XVD | 12 | 49.76 | 3 | 17 | 1 | 1 | 1 | 1 | 1 | 1 | 1 | 1 | 1 | 1 | 1 | 1 |
| 7BB6 | 9 | 70.79 | 46 | 9 | 27 | 47 | 58 | 30 | 50 | 59 | 31 | 45 | 59 | 8 | 18 | 31 |
| 7BB7 | 9 | 70.55 | 16 | 8 | 62 | 47 | 37 | 61 | 44 | 39 | 65 | 50 | 41 | 79 | 82 | 77 |
| 7DW9 | 9 | 61.83 | 2 | 1 | 1 | 1 | 2 | 1 | 1 | 2 | 1 | 1 | 1 | 1 | 1 | 1 |
| 7K2E | 7 | 48.6 | 1 | 14 | 19 | 6 | 7 | 23 | 8 | 8 | 22 | 9 | 6 | 4 | 1 | 2 |
| 7K2F | 7 | 48.76 | 5 | 1 | 1 | 1 | 1 | 1 | 1 | 1 | 1 | 1 | 1 | 1 | 1 | 1 |
| 7K2G | 7 | 47.51 | 13 | 8 | 5 | 3 | 2 | 6 | 3 | 2 | 7 | 3 | 2 | 11 | 11 | 5 |
| 7K2H | 7 | 50.96 | 1 | 1 | 8 | 2 | 2 | 9 | 3 | 2 | 9 | 3 | 2 | 6 | 2 | 2 |
| 7K2I | 7 | 52.4 | 3 | 1 | 1 | 2 | 2 | 1 | 2 | 2 | 1 | 2 | 3 | 3 | 3 | 2 |
| 7K2M | 7 | 50.72 | 1 | 1 | 1 | 4 | 11 | 1 | 2 | 10 | 1 | 2 | 8 | 1 | 1 | 2 |
| 7N0W | 14 | 62.2 | 1 | 8 | 1 | 1 | 1 | 1 | 1 | 1 | 1 | 1 | 1 | 1 | 1 | 1 |
| 7N43 | 17 | 72.08 | 2 | 18 | 24 | 25 | 29 | 24 | 23 | 29 | 23 | 22 | 25 | 27 | 24 | 19 |

*^a^* The order of the first conformation that meets the success criteria

**Table S12.** The ranks given by ADCP, Rosetta and MM/GB(PB)SA using ff14SBonlysc force field and implicit solvent model for dataset *II*

| PDB code | Peptide length | Polar interface (%) | ADCP | Rosetta | ff14SBonlysc implicit (ε_in_) | | | | | | | | | | | |
| --- | --- | --- | --- | --- | --- | --- | --- | --- | --- | --- | --- | --- | --- | --- | --- | --- |
|  |  |  |  |  | GBSA^HCT^ | | | GBSA^OBC1^ | | | GBSA^OBC2^ | | | PBSA | | |
|  |  |  |  |  | 1 | 2 | 4 | 1 | 2 | 4 | 1 | 2 | 4 | 1 | 2 | 4 |
| 1HQQ | 13 | 64.54 | 5*^a^* | 41 | 8 | 12 | 18 | 6 | 7 | 17 | 8 | 7 | 15 | 20 | 20 | 17 |
| 1HXL | 13 | 57.97 | 69 | 73 | 99 | 99 | 99 | 99 | 99 | 99 | 99 | 99 | 99 | 100 | 100 | 100 |
| 1HXZ | 13 | 59.95 | 10 | 32 | 3 | 4 | 5 | 3 | 4 | 5 | 2 | 4 | 4 | 3 | 10 | 18 |
| 1JK4 | 6 | 61.81 | 2 | 1 | 6 | 9 | 10 | 6 | 7 | 9 | 4 | 4 | 9 | 4 | 6 | 8 |
| 1SFI | 14 | 61.97 | 15 | 10 | 34 | 42 | 45 | 39 | 46 | 51 | 47 | 51 | 51 | 21 | 25 | 26 |
| 1SLD | 6 | 73.42 | 10 | 19 | 36 | 23 | 22 | 42 | 26 | 23 | 43 | 30 | 23 | 29 | 13 | 7 |
| 1SLE | 8 | 77.4 | 7 | 17 | 35 | 16 | 15 | 37 | 17 | 15 | 31 | 19 | 15 | 19 | 9 | 9 |
| 1SMF | 9 | 66.97 | 1 | 5 | 1 | 1 | 1 | 1 | 1 | 1 | 1 | 1 | 1 | 1 | 1 | 1 |
| 1VWB | 6 | 73.17 | 5 | 3 | 10 | 5 | 4 | 12 | 5 | 4 | 10 | 6 | 4 | 11 | 13 | 8 |
| 1VWC | 6 | 69.6 | 3 | 17 | 15 | 22 | 16 | 26 | 22 | 17 | 27 | 21 | 19 | 2 | 7 | 14 |
| 1VWD | 6 | 71.95 | 5 | 6 | 8 | 7 | 6 | 7 | 7 | 6 | 10 | 8 | 6 | 2 | 13 | 11 |
| 1VWE | 6 | 73.67 | 23 | 11 | 5 | 7 | 11 | 3 | 6 | 12 | 2 | 5 | 14 | 2 | 3 | 4 |
| 1VWM | 6 | 73.47 | 73 | 80 | 44 | 51 | 49 | 50 | 53 | 51 | 52 | 54 | 55 | 40 | 50 | 56 |
| 1VWN | 6 | 73.35 | 60 | 25 | 8 | 8 | 11 | 10 | 8 | 12 | 11 | 10 | 10 | 5 | 3 | 8 |
| 1YF4 | 9 | 50.68 | 1 | 16 | 1 | 1 | 1 | 1 | 1 | 2 | 1 | 1 | 2 | 1 | 1 | 1 |
| 2BR8 | 16 | 67.27 | 8 | 78 | 5 | 4 | 5 | 4 | 4 | 5 | 2 | 4 | 5 | 5 | 1 | 1 |
| 2C9T | 12 | 58.25 | 1 | 12 | 1 | 1 | 1 | 1 | 1 | 2 | 1 | 1 | 1 | 1 | 1 | 1 |
| 2NWN | 12 | 51.01 | 28 | 66 | 18 | 24 | 42 | 19 | 26 | 43 | 22 | 27 | 43 | 5 | 6 | 9 |
| 2UZ6 | 16 | 66.76 | 2 | 1 | 1 | 1 | 1 | 1 | 1 | 1 | 1 | 1 | 1 | 2 | 2 | 2 |
| 3AV9 | 8 | 61.29 | 7 | 1 | 6 | 7 | 9 | 6 | 7 | 8 | 6 | 7 | 7 | 1 | 1 | 1 |
| 3AVA | 8 | 61.58 | 9 | 4 | 3 | 7 | 16 | 6 | 10 | 17 | 8 | 12 | 19 | 3 | 3 | 3 |
| 3AVB | 8 | 61.38 | 2 | 2 | 2 | 1 | 1 | 2 | 1 | 1 | 2 | 1 | 1 | 12 | 17 | 17 |
| 3AVC | 8 | 62.3 | 33 | 3 | 2 | 1 | 2 | 2 | 1 | 2 | 2 | 1 | 3 | 12 | 27 | 4 |
| 3AVF | 8 | 59.7 | 14 | 1 | 2 | 1 | 1 | 1 | 1 | 1 | 1 | 1 | 1 | 1 | 1 | 3 |
| 3AVG | 8 | 61.83 | 4 | 3 | 2 | 3 | 6 | 2 | 3 | 5 | 2 | 3 | 5 | 1 | 2 | 4 |
| 3AVH | 8 | 62.45 | 2 | 1 | 2 | 1 | 2 | 2 | 1 | 2 | 2 | 2 | 3 | 2 | 3 | 4 |
| 3AVI | 8 | 63.35 | 2 | 5 | 3 | 3 | 2 | 3 | 3 | 1 | 2 | 2 | 1 | 4 | 2 | 3 |
| 3AVJ | 8 | 64.8 | 3 | 6 | 5 | 4 | 2 | 5 | 4 | 2 | 5 | 4 | 2 | 2 | 2 | 2 |
| 3AVK | 8 | 63.12 | 1 | 1 | 1 | 1 | 1 | 1 | 1 | 1 | 1 | 1 | 1 | 1 | 1 | 1 |
| 3AVL | 8 | 61.54 | 11 | 1 | 1 | 2 | 3 | 1 | 2 | 3 | 1 | 2 | 3 | 1 | 2 | 3 |
| 3AVM | 8 | 61.26 | 2 | 1 | 2 | 1 | 1 | 4 | 1 | 1 | 4 | 1 | 1 | 3 | 2 | 2 |
| 3AVN | 8 | 61.76 | 14 | 1 | 1 | 6 | 9 | 1 | 7 | 9 | 1 | 4 | 9 | 2 | 2 | 3 |
| 3M61 | 12 | 49.48 | 17 | 12 | 1 | 1 | 1 | 1 | 1 | 2 | 1 | 1 | 1 | 8 | 2 | 1 |
| 3P72 | 11 | 62.62 | 3 | 7 | 1 | 24 | 33 | 1 | 24 | 33 | 1 | 18 | 33 | 7 | 10 | 14 |
| 3P8F | 14 | 65.48 | 3 | 28 | 2 | 2 | 3 | 2 | 2 | 3 | 2 | 2 | 3 | 1 | 1 | 1 |
| 3WNE | 6 | 63.78 | 1 | 1 | 1 | 1 | 1 | 1 | 1 | 1 | 1 | 1 | 1 | 1 | 1 | 1 |
| 3WNF | 6 | 65.77 | 4 | 10 | 8 | 7 | 5 | 4 | 5 | 4 | 4 | 5 | 3 | 6 | 5 | 2 |
| 3ZGC | 7 | 48.78 | 2 | 11 | 6 | 6 | 10 | 9 | 7 | 9 | 11 | 9 | 10 | 2 | 3 | 5 |
| 4EZ1 | 13 | 71.56 | 1 | 7 | 31 | 46 | 58 | 34 | 47 | 60 | 29 | 42 | 57 | 96 | 94 | 89 |
| 4GLY | 13 | 48.12 | 3 | 12 | 2 | 9 | 6 | 2 | 5 | 6 | 2 | 5 | 6 | 1 | 1 | 1 |
| 4K1E | 14 | 65.55 | 19 | 60 | 2 | 4 | 7 | 2 | 4 | 7 | 2 | 5 | 7 | 2 | 2 | 1 |
| 4KEL | 14 | 65.36 | 16 | 19 | 5 | 5 | 8 | 5 | 7 | 8 | 6 | 8 | 8 | 6 | 3 | 4 |
| 4OU3 | 6 | 42.09 | 7 | 19 | 55 | 58 | 19 | 56 | 58 | 23 | 56 | 59 | 27 | 83 | 84 | 75 |
| 4X1Q | 10 | 56.36 | 3 | 26 | 1 | 1 | 1 | 1 | 1 | 1 | 1 | 1 | 1 | 1 | 1 | 1 |
| 4XOJ | 13 | 60.96 | 3 | 7 | 1 | 1 | 1 | 1 | 1 | 1 | 1 | 1 | 1 | 1 | 1 | 1 |
| 4Z09 | 13 | 65.07 | 3 | 9 | 1 | 3 | 6 | 1 | 3 | 6 | 1 | 4 | 6 | 1 | 1 | 1 |
| 4ZHL | 10 | 59.9 | 6 | 10 | 1 | 1 | 1 | 1 | 1 | 1 | 1 | 1 | 1 | 1 | 1 | 1 |
| 4ZKS | 12 | 49.14 | 49 | 78 | 6 | 4 | 4 | 6 | 5 | 5 | 6 | 5 | 5 | 14 | 10 | 5 |
| 5CO5 | 16 | 62.1 | 2 | 15 | 8 | 15 | 22 | 6 | 13 | 21 | 6 | 13 | 21 | 1 | 4 | 9 |
| 5DI8 | 13 | 65.33 | 2 | 8 | 4 | 2 | 2 | 5 | 1 | 2 | 5 | 2 | 2 | 5 | 1 | 1 |
| 5DJ0 | 13 | 65.82 | 67 | 11 | 66 | 72 | 70 | 65 | 72 | 69 | 69 | 73 | 72 | 17 | 22 | 31 |
| 5DJ6 | 13 | 65 | 5 | 15 | 46 | 34 | 29 | 38 | 31 | 27 | 42 | 32 | 29 | 3 | 1 | 2 |
| 5DJ8 | 13 | 66.31 | 24 | 15 | 6 | 6 | 6 | 8 | 6 | 6 | 6 | 7 | 6 | 9 | 5 | 5 |
| 5DJC | 13 | 63.72 | 4 | 18 | 8 | 4 | 3 | 6 | 4 | 2 | 5 | 4 | 3 | 5 | 5 | 6 |
| 5DJX | 13 | 66.91 | 25 | 2 | 48 | 62 | 69 | 42 | 57 | 69 | 43 | 58 | 68 | 33 | 48 | 56 |
| 5DJY | 13 | 63.52 | 26 | 14 | 33 | 33 | 30 | 30 | 32 | 30 | 34 | 36 | 30 | 3 | 5 | 8 |
| 5DJZ | 13 | 64.62 | 2 | 27 | 6 | 5 | 5 | 6 | 5 | 5 | 5 | 4 | 5 | 3 | 3 | 4 |
| 5DK0 | 13 | 66.71 | 1 | 49 | 1 | 4 | 4 | 1 | 4 | 4 | 1 | 4 | 4 | 1 | 1 | 2 |
| 5DVL | 13 | 69.61 | 2 | 37 | 8 | 7 | 7 | 8 | 5 | 7 | 12 | 6 | 7 | 16 | 2 | 1 |
| 5DVN | 13 | 70.37 | 42 | 9 | 7 | 11 | 18 | 13 | 13 | 20 | 15 | 11 | 18 | 32 | 48 | 59 |
| 5EOC | 13 | 61.52 | 49 | 46 | 94 | 96 | 96 | 92 | 96 | 96 | 88 | 96 | 96 | 42 | 55 | 71 |
| 5GRD | 10 | 59.7 | 2 | 1 | 1 | 1 | 1 | 1 | 1 | 1 | 1 | 1 | 1 | 1 | 1 | 1 |
| 5GRG | 4 | 54.93 | 1 | 1 | 1 | 1 | 1 | 1 | 1 | 1 | 1 | 1 | 1 | 1 | 1 | 1 |
| 5JZU | 13 | 57.73 | 9 | 6 | 3 | 6 | 9 | 2 | 6 | 9 | 2 | 5 | 8 | 14 | 16 | 18 |
| 5TU6 | 7 | 69.69 | 80 | 91 | 32 | 22 | 18 | 41 | 26 | 20 | 57 | 35 | 25 | 43 | 27 | 19 |
| 5XN3 | 8 | 49.33 | 5 | 1 | 1 | 1 | 7 | 1 | 1 | 7 | 1 | 1 | 7 | 1 | 1 | 1 |
| 6BVH | 14 | 58.18 | 64 | 30 | 4 | 10 | 17 | 3 | 10 | 17 | 3 | 10 | 17 | 2 | 3 | 5 |
| 6D3Y | 14 | 58.28 | 2 | 25 | 1 | 1 | 3 | 1 | 1 | 3 | 1 | 1 | 3 | 1 | 1 | 1 |
| 6D3Z | 14 | 62.3 | 2 | 28 | 1 | 1 | 2 | 1 | 1 | 2 | 1 | 1 | 2 | 1 | 1 | 1 |
| 6XVD | 12 | 49.76 | 3 | 17 | 1 | 1 | 1 | 1 | 1 | 2 | 1 | 1 | 2 | 1 | 1 | 1 |
| 7BB6 | 9 | 70.79 | 46 | 9 | 32 | 40 | 52 | 28 | 39 | 52 | 27 | 36 | 49 | 27 | 30 | 32 |
| 7BB7 | 9 | 70.55 | 16 | 8 | 16 | 21 | 30 | 18 | 25 | 35 | 17 | 25 | 34 | 13 | 17 | 21 |
| 7DW9 | 9 | 61.83 | 2 | 1 | 1 | 1 | 1 | 1 | 1 | 1 | 1 | 1 | 1 | 1 | 1 | 1 |
| 7K2E | 7 | 48.6 | 1 | 14 | 8 | 8 | 10 | 5 | 6 | 10 | 7 | 5 | 9 | 4 | 2 | 3 |
| 7K2F | 7 | 48.76 | 5 | 1 | 1 | 1 | 1 | 1 | 1 | 1 | 1 | 1 | 1 | 1 | 2 | 3 |
| 7K2G | 7 | 47.51 | 13 | 8 | 3 | 1 | 1 | 2 | 1 | 1 | 2 | 1 | 1 | 4 | 8 | 4 |
| 7K2H | 7 | 50.96 | 1 | 1 | 11 | 8 | 10 | 14 | 10 | 11 | 15 | 11 | 12 | 12 | 11 | 7 |
| 7K2I | 7 | 52.4 | 3 | 1 | 4 | 3 | 2 | 4 | 2 | 2 | 6 | 2 | 2 | 5 | 3 | 3 |
| 7K2M | 7 | 50.72 | 1 | 1 | 1 | 4 | 6 | 1 | 4 | 5 | 2 | 3 | 5 | 1 | 1 | 2 |
| 7N0W | 14 | 62.2 | 1 | 8 | 1 | 1 | 1 | 1 | 1 | 1 | 1 | 1 | 1 | 1 | 1 | 1 |
| 7N43 | 17 | 72.08 | 2 | 18 | 62 | 48 | 42 | 66 | 48 | 41 | 70 | 52 | 41 | 7 | 24 | 48 |

*^a^* The order of the first conformation that meets the success criteria

**Table S13.** The ranks given by ADCP, Rosetta and MM/GB(PB)SA using ff14SBonlysc force field and explicit solvent model for dataset *II*

| PDB code | Peptide length | Polar interface (%) | ADCP | Rosetta | ff14SBonlysc explicit (ε_in_) | | | | | | | | | | | |
| --- | --- | --- | --- | --- | --- | --- | --- | --- | --- | --- | --- | --- | --- | --- | --- | --- |
|  |  |  |  |  | GBSA^HCT^ | | | GBSA^OBC1^ | | | GBSA^OBC2^ | | | PBSA | | |
|  |  |  |  |  | 1 | 2 | 4 | 1 | 2 | 4 | 1 | 2 | 4 | 1 | 2 | 4 |
| 1HQQ | 13 | 64.54 | 5*^a^* | 41 | 7 | 10 | 12 | 4 | 6 | 10 | 5 | 5 | 10 | 8 | 5 | 7 |
| 1HXL | 13 | 57.97 | 69 | 73 | 48 | 24 | 17 | 48 | 28 | 17 | 46 | 28 | 16 | 51 | 43 | 37 |
| 1HXZ | 13 | 59.95 | 10 | 32 | 15 | 29 | 32 | 12 | 34 | 36 | 10 | 34 | 35 | 13 | 34 | 35 |
| 1JK4 | 6 | 61.81 | 2 | 1 | 6 | 11 | 11 | 6 | 9 | 11 | 5 | 8 | 11 | 5 | 9 | 8 |
| 1SFI | 14 | 61.97 | 15 | 10 | 37 | 40 | 43 | 44 | 42 | 46 | 54 | 51 | 48 | 18 | 21 | 25 |
| 1SLD | 6 | 73.42 | 10 | 19 | 47 | 18 | 5 | 50 | 19 | 5 | 50 | 23 | 9 | 21 | 26 | 12 |
| 1SLE | 8 | 77.4 | 7 | 17 | 18 | 28 | 22 | 20 | 31 | 23 | 19 | 32 | 23 | 7 | 15 | 33 |
| 1SMF | 9 | 66.97 | 1 | 5 | 1 | 1 | 1 | 1 | 1 | 1 | 1 | 1 | 1 | 1 | 1 | 1 |
| 1VWB | 6 | 73.17 | 5 | 3 | 3 | 9 | 9 | 4 | 9 | 9 | 4 | 9 | 10 | 2 | 5 | 9 |
| 1VWC | 6 | 69.6 | 3 | 17 | 33 | 9 | 7 | 43 | 12 | 7 | 46 | 16 | 8 | 24 | 45 | 18 |
| 1VWD | 6 | 71.95 | 5 | 6 | 6 | 5 | 5 | 6 | 5 | 5 | 8 | 6 | 5 | 2 | 2 | 8 |
| 1VWE | 6 | 73.67 | 23 | 11 | 8 | 9 | 14 | 12 | 13 | 14 | 13 | 14 | 14 | 3 | 1 | 3 |
| 1VWM | 6 | 73.47 | 73 | 80 | 23 | 39 | 48 | 32 | 43 | 52 | 39 | 45 | 53 | 33 | 52 | 70 |
| 1VWN | 6 | 73.35 | 60 | 25 | 7 | 16 | 20 | 8 | 18 | 22 | 8 | 16 | 24 | 1 | 1 | 2 |
| 1YF4 | 9 | 50.68 | 1 | 16 | 1 | 1 | 2 | 1 | 2 | 2 | 1 | 2 | 2 | 1 | 1 | 1 |
| 2BR8 | 16 | 67.27 | 8 | 78 | 4 | 3 | 2 | 1 | 1 | 2 | 1 | 1 | 2 | 7 | 1 | 1 |
| 2C9T | 12 | 58.25 | 1 | 12 | 1 | 1 | 2 | 1 | 1 | 2 | 1 | 1 | 2 | 1 | 1 | 1 |
| 2NWN | 12 | 51.01 | 28 | 66 | 7 | 8 | 10 | 8 | 9 | 10 | 9 | 9 | 11 | 6 | 5 | 5 |
| 2UZ6 | 16 | 66.76 | 2 | 1 | 1 | 1 | 1 | 1 | 1 | 1 | 1 | 1 | 1 | 1 | 1 | 1 |
| 3AV9 | 8 | 61.29 | 7 | 1 | 16 | 15 | 7 | 15 | 15 | 7 | 15 | 16 | 8 | 14 | 14 | 14 |
| 3AVA | 8 | 61.58 | 9 | 4 | 5 | 6 | 11 | 6 | 6 | 11 | 11 | 9 | 11 | 4 | 5 | 4 |
| 3AVB | 8 | 61.38 | 2 | 2 | 16 | 16 | 13 | 17 | 16 | 13 | 16 | 16 | 12 | 13 | 23 | 33 |
| 3AVC | 8 | 62.3 | 33 | 3 | 7 | 5 | 4 | 7 | 9 | 4 | 9 | 12 | 6 | 9 | 11 | 5 |
| 3AVF | 8 | 59.7 | 14 | 1 | 3 | 6 | 9 | 3 | 7 | 10 | 3 | 7 | 10 | 1 | 3 | 6 |
| 3AVG | 8 | 61.83 | 4 | 3 | 3 | 4 | 6 | 3 | 2 | 6 | 3 | 4 | 6 | 2 | 3 | 4 |
| 3AVH | 8 | 62.45 | 2 | 1 | 2 | 1 | 1 | 2 | 1 | 1 | 3 | 2 | 1 | 1 | 3 | 3 |
| 3AVI | 8 | 63.35 | 2 | 5 | 1 | 1 | 6 | 1 | 1 | 5 | 1 | 1 | 3 | 1 | 1 | 2 |
| 3AVJ | 8 | 64.8 | 3 | 6 | 1 | 1 | 2 | 1 | 1 | 2 | 1 | 1 | 2 | 2 | 2 | 2 |
| 3AVK | 8 | 63.12 | 1 | 1 | 1 | 1 | 1 | 1 | 1 | 1 | 1 | 1 | 1 | 1 | 1 | 1 |
| 3AVL | 8 | 61.54 | 11 | 1 | 9 | 10 | 14 | 8 | 10 | 14 | 8 | 10 | 13 | 4 | 3 | 4 |
| 3AVM | 8 | 61.26 | 2 | 1 | 4 | 4 | 2 | 5 | 4 | 2 | 6 | 4 | 2 | 3 | 1 | 1 |
| 3AVN | 8 | 61.76 | 14 | 1 | 9 | 6 | 11 | 9 | 7 | 13 | 9 | 7 | 13 | 12 | 13 | 13 |
| 3M61 | 12 | 49.48 | 17 | 12 | 2 | 6 | 15 | 2 | 6 | 15 | 2 | 4 | 14 | 3 | 3 | 10 |
| 3P72 | 11 | 62.62 | 3 | 7 | 11 | 40 | 62 | 9 | 36 | 61 | 7 | 32 | 60 | 5 | 5 | 16 |
| 3P8F | 14 | 65.48 | 3 | 28 | 2 | 2 | 2 | 2 | 2 | 2 | 2 | 2 | 2 | 1 | 1 | 1 |
| 3WNE | 6 | 63.78 | 1 | 1 | 1 | 1 | 1 | 1 | 1 | 1 | 1 | 1 | 1 | 1 | 1 | 1 |
| 3WNF | 6 | 65.77 | 4 | 10 | 10 | 7 | 7 | 5 | 7 | 6 | 3 | 5 | 6 | 6 | 4 | 7 |
| 3ZGC | 7 | 48.78 | 2 | 11 | 8 | 8 | 13 | 8 | 10 | 15 | 10 | 11 | 18 | 11 | 12 | 14 |
| 4EZ1 | 13 | 71.56 | 1 | 7 | 48 | 40 | 42 | 48 | 38 | 40 | 45 | 39 | 39 | 91 | 89 | 78 |
| 4GLY | 13 | 48.12 | 3 | 12 | 4 | 4 | 9 | 3 | 4 | 9 | 4 | 4 | 9 | 1 | 1 | 1 |
| 4K1E | 14 | 65.55 | 19 | 60 | 2 | 8 | 15 | 3 | 12 | 17 | 3 | 12 | 17 | 1 | 1 | 1 |
| 4KEL | 14 | 65.36 | 16 | 19 | 8 | 17 | 22 | 8 | 17 | 23 | 12 | 18 | 23 | 7 | 8 | 12 |
| 4OU3 | 6 | 42.09 | 7 | 19 | 67 | 58 | 33 | 71 | 61 | 36 | 76 | 62 | 38 | 72 | 76 | 76 |
| 4X1Q | 10 | 56.36 | 3 | 26 | 2 | 2 | 2 | 1 | 2 | 2 | 1 | 1 | 2 | 1 | 1 | 1 |
| 4XOJ | 13 | 60.96 | 3 | 7 | 1 | 1 | 1 | 1 | 1 | 1 | 1 | 1 | 1 | 1 | 1 | 1 |
| 4Z09 | 13 | 65.07 | 3 | 9 | 1 | 1 | 14 | 1 | 2 | 15 | 1 | 2 | 13 | 1 | 1 | 3 |
| 4ZHL | 10 | 59.9 | 6 | 10 | 1 | 1 | 2 | 1 | 1 | 1 | 1 | 1 | 1 | 1 | 1 | 1 |
| 4ZKS | 12 | 49.14 | 49 | 78 | 1 | 2 | 6 | 1 | 1 | 6 | 1 | 1 | 5 | 2 | 1 | 1 |
| 5CO5 | 16 | 62.1 | 2 | 15 | 16 | 19 | 21 | 15 | 17 | 20 | 14 | 17 | 20 | 1 | 1 | 2 |
| 5DI8 | 13 | 65.33 | 2 | 8 | 7 | 3 | 3 | 6 | 3 | 3 | 5 | 4 | 4 | 1 | 1 | 1 |
| 5DJ0 | 13 | 65.82 | 67 | 11 | 27 | 36 | 42 | 27 | 35 | 44 | 32 | 36 | 44 | 52 | 55 | 56 |
| 5DJ6 | 13 | 65 | 5 | 15 | 19 | 12 | 11 | 21 | 11 | 11 | 27 | 14 | 12 | 4 | 3 | 5 |
| 5DJ8 | 13 | 66.31 | 24 | 15 | 8 | 7 | 5 | 10 | 7 | 6 | 10 | 8 | 5 | 9 | 4 | 3 |
| 5DJC | 13 | 63.72 | 4 | 18 | 2 | 4 | 4 | 2 | 3 | 5 | 2 | 3 | 4 | 22 | 17 | 16 |
| 5DJX | 13 | 66.91 | 25 | 2 | 63 | 74 | 80 | 61 | 71 | 80 | 62 | 73 | 79 | 41 | 52 | 68 |
| 5DJY | 13 | 63.52 | 26 | 14 | 19 | 27 | 30 | 21 | 26 | 30 | 23 | 24 | 31 | 4 | 6 | 16 |
| 5DJZ | 13 | 64.62 | 2 | 27 | 5 | 2 | 4 | 5 | 2 | 4 | 5 | 3 | 4 | 1 | 1 | 1 |
| 5DK0 | 13 | 66.71 | 1 | 49 | 3 | 1 | 1 | 3 | 1 | 1 | 3 | 2 | 1 | 1 | 2 | 2 |
| 5DVL | 13 | 69.61 | 2 | 37 | 9 | 5 | 6 | 9 | 4 | 4 | 10 | 5 | 4 | 3 | 2 | 1 |
| 5DVN | 13 | 70.37 | 42 | 9 | 1 | 2 | 3 | 1 | 3 | 3 | 1 | 3 | 3 | 2 | 1 | 2 |
| 5EOC | 13 | 61.52 | 49 | 46 | 81 | 84 | 85 | 80 | 83 | 85 | 81 | 83 | 85 | 37 | 38 | 45 |
| 5GRD | 10 | 59.7 | 2 | 1 | 1 | 1 | 1 | 1 | 1 | 1 | 1 | 1 | 1 | 2 | 1 | 1 |
| 5GRG | 4 | 54.93 | 1 | 1 | 1 | 1 | 1 | 1 | 1 | 1 | 1 | 1 | 1 | 1 | 1 | 1 |
| 5JZU | 13 | 57.73 | 9 | 6 | 1 | 2 | 8 | 1 | 1 | 8 | 1 | 1 | 6 | 7 | 8 | 9 |
| 5TU6 | 7 | 69.69 | 80 | 91 | 52 | 42 | 33 | 55 | 43 | 34 | 59 | 48 | 38 | 62 | 62 | 59 |
| 5XN3 | 8 | 49.33 | 5 | 1 | 1 | 3 | 9 | 1 | 3 | 9 | 1 | 3 | 8 | 1 | 2 | 3 |
| 6BVH | 14 | 58.18 | 64 | 30 | 1 | 4 | 10 | 1 | 3 | 9 | 1 | 2 | 9 | 1 | 1 | 1 |
| 6D3Y | 14 | 58.28 | 2 | 25 | 1 | 4 | 14 | 1 | 4 | 15 | 1 | 2 | 15 | 1 | 1 | 1 |
| 6D3Z | 14 | 62.3 | 2 | 28 | 3 | 5 | 15 | 3 | 8 | 15 | 4 | 6 | 14 | 1 | 1 | 4 |
| 6XVD | 12 | 49.76 | 3 | 17 | 1 | 1 | 1 | 1 | 1 | 1 | 1 | 1 | 1 | 1 | 1 | 1 |
| 7BB6 | 9 | 70.79 | 46 | 9 | 17 | 40 | 67 | 13 | 40 | 67 | 9 | 33 | 62 | 3 | 15 | 40 |
| 7BB7 | 9 | 70.55 | 16 | 8 | 46 | 39 | 42 | 48 | 42 | 41 | 50 | 43 | 40 | 26 | 31 | 41 |
| 7DW9 | 9 | 61.83 | 2 | 1 | 1 | 1 | 2 | 1 | 1 | 1 | 1 | 1 | 1 | 1 | 1 | 1 |
| 7K2E | 7 | 48.6 | 1 | 14 | 18 | 18 | 8 | 20 | 20 | 9 | 14 | 14 | 13 | 9 | 8 | 10 |
| 7K2F | 7 | 48.76 | 5 | 1 | 1 | 1 | 1 | 1 | 1 | 1 | 1 | 1 | 1 | 2 | 3 | 3 |
| 7K2G | 7 | 47.51 | 13 | 8 | 1 | 1 | 1 | 1 | 1 | 1 | 1 | 1 | 1 | 4 | 1 | 1 |
| 7K2H | 7 | 50.96 | 1 | 1 | 5 | 2 | 3 | 5 | 2 | 3 | 6 | 2 | 2 | 9 | 3 | 3 |
| 7K2I | 7 | 52.4 | 3 | 1 | 2 | 2 | 2 | 2 | 2 | 3 | 2 | 2 | 3 | 3 | 3 | 6 |
| 7K2M | 7 | 50.72 | 1 | 1 | 2 | 2 | 5 | 4 | 2 | 5 | 4 | 3 | 4 | 2 | 1 | 1 |
| 7N0W | 14 | 62.2 | 1 | 8 | 1 | 1 | 1 | 1 | 1 | 1 | 1 | 1 | 1 | 1 | 1 | 1 |
| 7N43 | 17 | 72.08 | 2 | 18 | 4 | 5 | 11 | 4 | 5 | 10 | 4 | 5 | 10 | 14 | 5 | 5 |

*^a^* The order of the first conformation that meets the success criteria


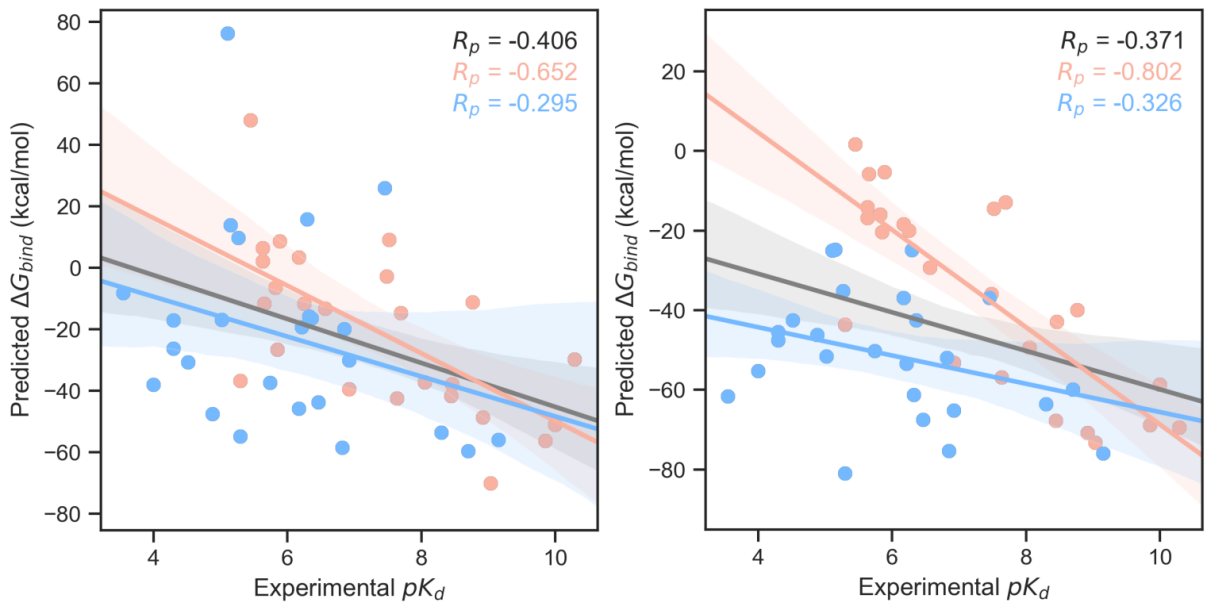


**Figure S1.** Representative scatter plots of binding affinity predictions for implicit and explicit solvent models. Binding affinities were predicted using the ff14SB force field and the MM/PBSA method on energy-minimized structures (internal dielectric constant = 1). Results obtained with the implicit and explicit solvent models are shown in the left and right panels, respectively. Pink and blue dots represent complexes with high and low polarity, respectively. *R_p_* are displayed in the upper right corner of each panel: black corresponds to the entire Dataset *I*, pink to the high-polarity subset, and blue to the low-polarity subset.


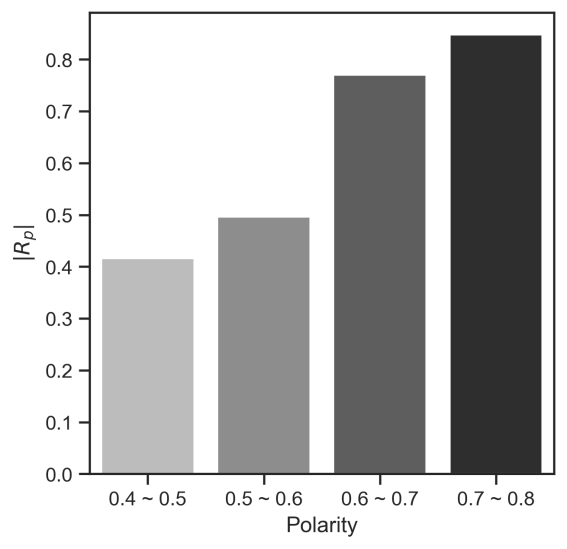

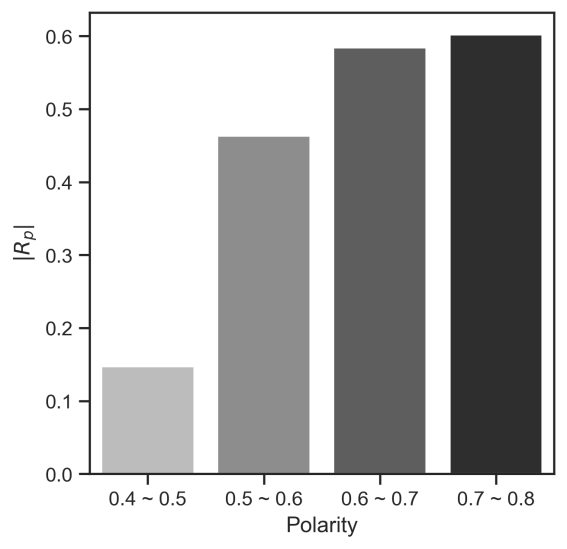


**Figure S2.** Variation of prediction accuracy with interface polarity in P–cp complexes. Bar plots show the absolute Pearson correlation coefficients between the predicted (ΔG_predicted_) and experimental (ΔG_experimental_) binding affinities across different interface polarity intervals (0.4–0.5, 0.5–0.6, 0.6–0.7, and 0.7–0.8). The analysis was performed using the ff14SB force field with the MM/PBSA method on minimized structures (internal dielectric constant = 1). The left and right panels correspond to the results obtained with implicit and explicit solvent models, respectively.


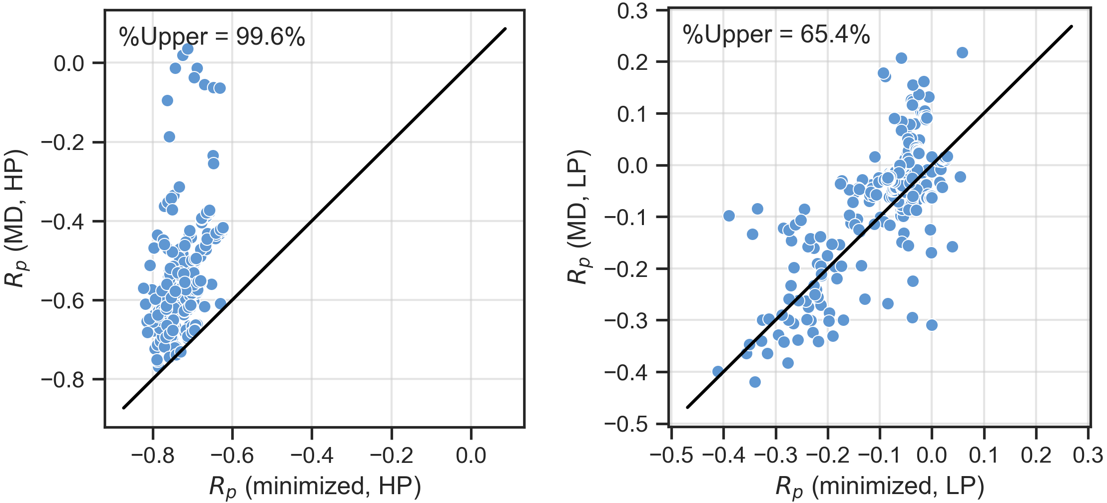


**Figure S3.** Comparison of Pearson correlation coefficients (*R_p_*) between the experimentally determined pK_d_ and the predicted binding affinities calculated by MM/PBSA(GBSA) using the energy-optimized structures (minimized) and the structures derived from the 5ns MD simulations (MD). The left and right panels correspond to the high polarity (HP) and low polarity (LP) subsets, respectively. The percentages denoted as %Upper represent the proportion of points above the diagonal among all points. A higher %Upper value indicates a larger fraction of cases where *R_p_* values from minimized structures are better than those from MD.


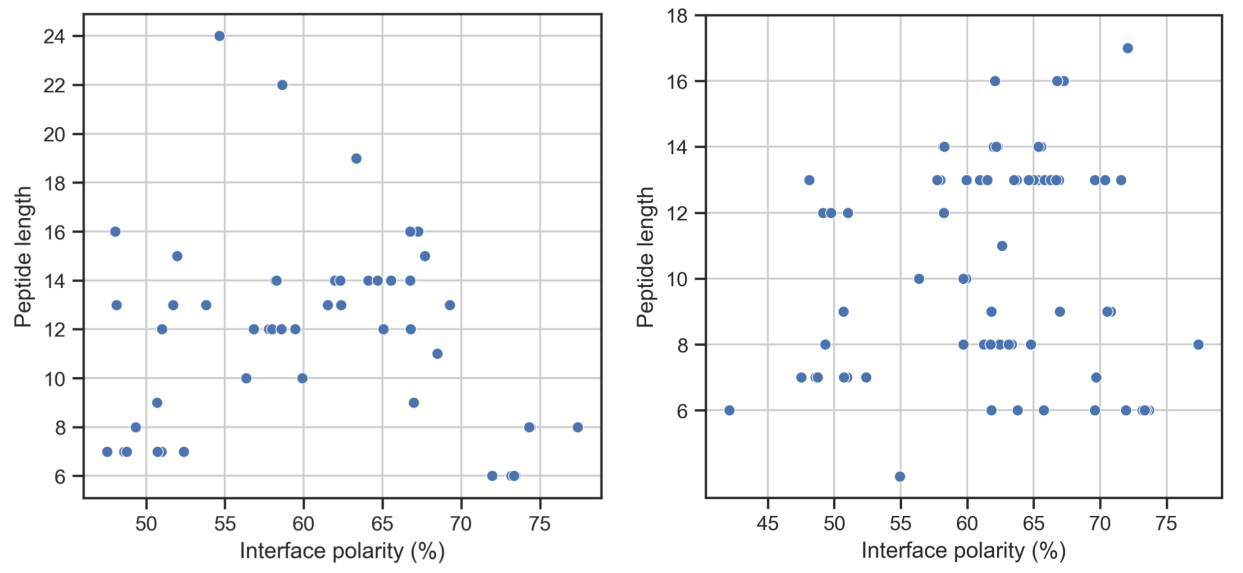


**Figure S4.** Scatter plot of protein–cyclic peptide (P–cp) complexes by interface polarity and peptide chain length. The left and right panels correspond to Dataset *I* and Dataset *II*, respectively.


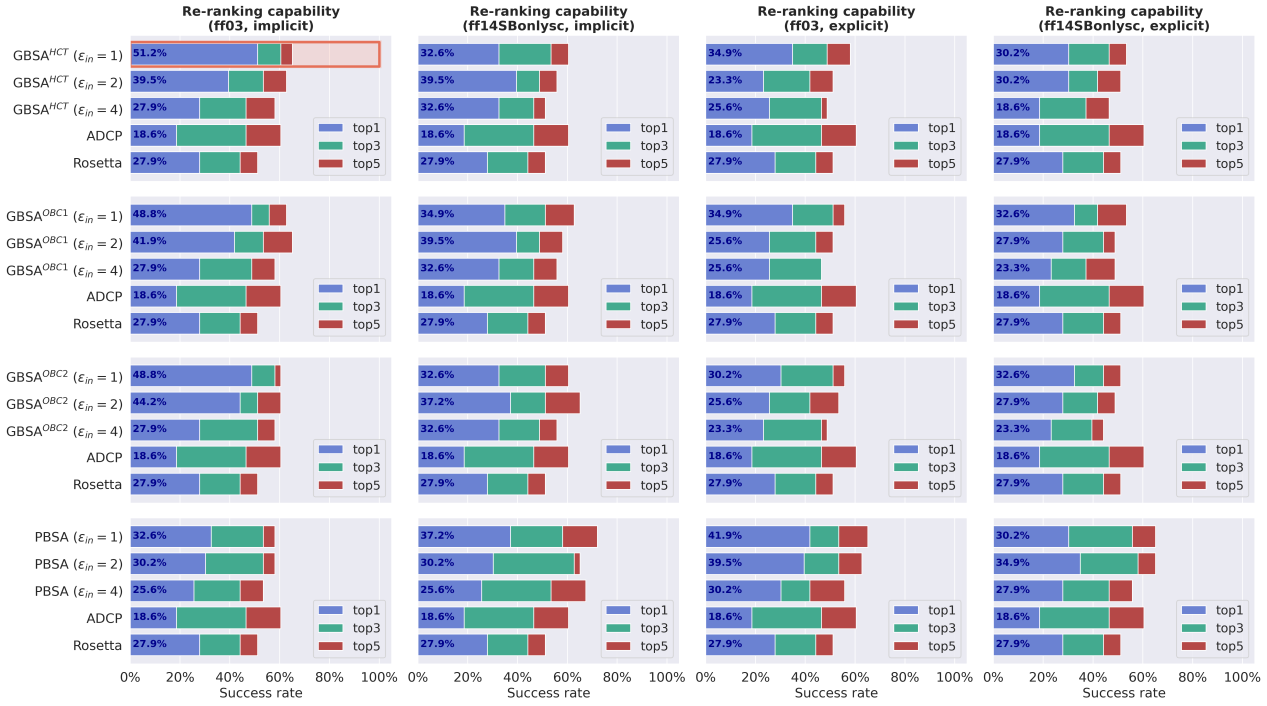


**Figure S5.** Success rates of ADCP, Rosetta and different MM/PBSA and MM/GBSA protocols for the short subset.


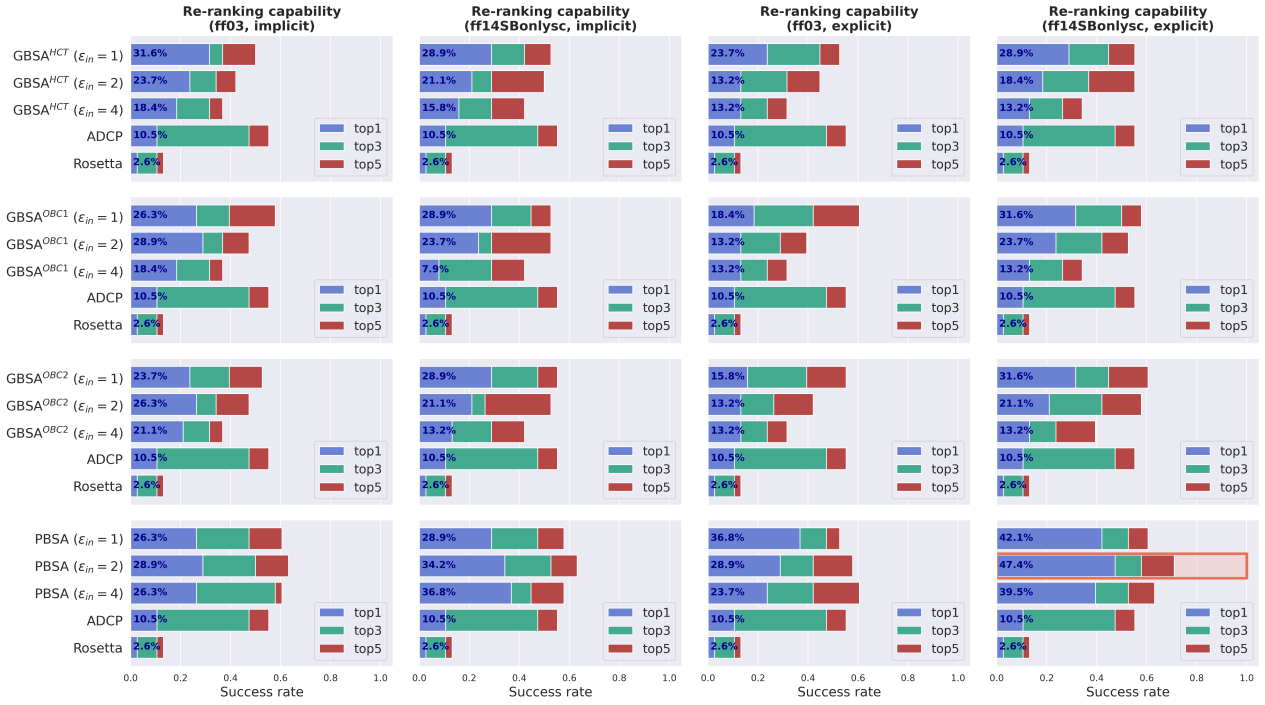


**Figure S6.** Success rates of ADCP, Rosetta and different MM/PBSA and MM/GBSA protocols for the long subset.


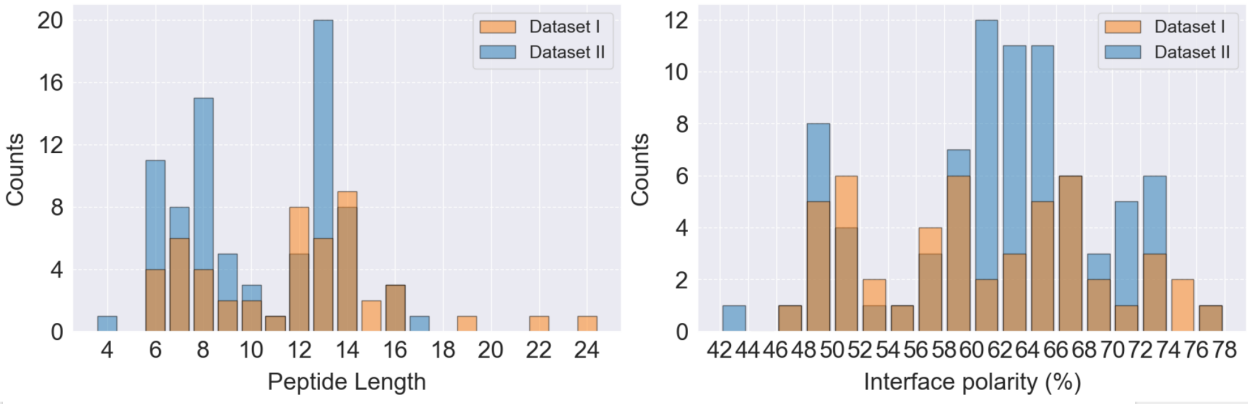


**Figure S7.** Histogram of subsets with different peptide chain length (left) and polarity (right) in Dataset *I* and Dataset *II*.
